# A Novel Nuclear Protein Complex Controlling the Expression of Developmentally Regulated Genes in *Toxoplasma Gondii*

**DOI:** 10.1002/advs.202412000

**Published:** 2024-12-24

**Authors:** Lilan Xue, Jingwen Zhang, Lihong Zhang, Fuqiang Fan, Xiaoyan Yin, Hengrui Tian, Bang Shen

**Affiliations:** ^1^ State Key Laboratory of Agricultural Microbiology College of Veterinary Medicine Huazhong Agricultural University Wuhan 430070 P. R. China; ^2^ Hubei Hongshan Laboratory Wuhan 430070 P. R. China; ^3^ Shenzhen Institute of Nutrition and Health Huazhong Agricultural University Shenzhen 518000 P. R. China; ^4^ Shenzhen Branch, Guangdong Laboratory for Lingnan Modern Agriculture, Genome Analysis Laboratory of the Ministry of Agriculture Agricultural Genomics Institute at Shenzhen, Chinese Academy of Agricultural Sciences Shenzhen 518000 P. R. China

**Keywords:** AP2 transcription factor, bradyzoite, epigenetic factor, HDAC3, merozoite

## Abstract

*Toxoplasma gondii* is a ubiquitous protozoan parasite with a complex life cycle containing multiple developmental stages. The parasites have distinct gene expression patterns at different stages to enable stage specific life activities, but the underlying regulatory mechanisms are largely unknown. In this study, a nuclear complex is identified that controls the expression of developmentally regulated genes. This complex consists of the AP2 family transcription factor AP2XII‐5, the epigenetic factors MORC and HDAC3, as well as a novel AP2XII‐5 interacting protein 1 (AIP1) that stabilizes this complex. At the tachyzoite stage when the parasites proliferate rapidly by asexual endodyogeny, AP2XII‐5 binds to the promoter regions of developmentally activated genes and recruits MORC and HDAC3 to suppress their expression. When sexual commitment and merozoite development are triggered, the abundance of AP2XII‐5 decreases and its suppression on target genes is relieved. In contrast to MORC and HDAC3, which regulate *Toxoplasma* development but are also essential for tachyzoite growth, AP2XII‐5 and AIP1 are dispensable for tachyzoite proliferation in vitro. These data suggest that while MORC and HDAC3 have broad regulatory activities, forming a complex with AP2XII‐5 and AIP1 enables them to specifically regulate gene expression during development.

## Graphical abstract

*Toxoplasma gondii* is a ubiquitous pathogen infecting billions of people and numerous animals. It has a complex life cycle with multiple developmental stages that facilitate its transmission. This study reports the key role of an AP2 family transcription factor called AP2XII‐5, by forming a complex with other nuclear factors and epigenetic factors, in regulating the expression of developmental genes.

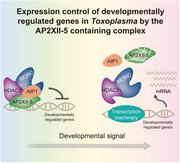

## 1 Introduction

*Toxoplasma gondii* is a globally prevalent zoonotic pathogen, infecting nearly one‐third of the human population.^[^ ^1^ ^]^ The parasite has a complex life cycle that is characterized by multiple developmental stages, which significantly contributes to its transmission and pathogenesis. The ability of *T. gondii* to interconvert between rapidly replicating tachyzoites and semi‐dormant bradyzoites enables it to transmit and persist among intermediate hosts.^[^ ^2^ ^]^ Sexual reproduction within the definitive feline hosts produces environmentally resistant oocysts that are the major source of infection for intermediate hosts. Sexual reproduction occurs in the intestinal epithelial cells of cats and it is a complicated process involving multiple stages.^[^ ^3^ ^]^ Infecting cats with bradyzoites is the most efficient and productive way in promoting oocyst production.^[^ ^4^ ^]^ Ingested bradyzoites are first converted to merozoites that replicate rapidly by asexual merogony. Then, some merozoites differentiate to male and female gametocytes, which subsequently generate male and female gametes that are fertilized to produce oocysts.^[^ ^5^ ^]^ Except the interconversion between tachyzoites and bradyzoites, which is a unique aspect of the *T. gondii* life cycle, the development of the *Toxoplasma* parasite is unidirectional, like other apicomplexan parasites.^[^ ^6^ ^]^

Changes in the parasitic environments are a key driven force for the parasites to alter the developmental programs. For example, stress conditions like nutrient starvation often trigger the transition of tachyzoites to bradyzoites.^[^ ^7^ ^]^ CO_2_ starvation, along with alkaline medium, is commonly used to induce bradyzoite formation in vitro.^[^ ^8^ ^]^ Consequently, the parasites displayed distinct gene expression patterns at different developmental stages, to facilitate the adaptation to stage specific life activities. Numerous studies have looked into the gene expression differences across different life cycle stages.^[^ ^9^ ^]^ These studies revealed many stage‐specific genes that can be used as developmental markers, such as SAG1 (Surface antigen 1) for tachyzoites, BAG1 for bradyzoites, and GRA11B for merozoites.^[^ ^10^ ^]^ In addition, these transcriptomic analyses suggest that the gene expression must be fine‐tuned to achieve a “just‐in‐time” pattern, which ensures the expression of the correct set of genes when they are needed.

While the gene expression differences across developmental stages have been extensively documented, the underlying regulatory mechanisms are still poorly understood. One feature of the apicomplexan parasites is the low ratio of known transcription factors (TFs) to protein‐coding genes, compared to model organisms like yeasts, fruit flies, and mice. Many conserved canonical TFs like Myb and C2H2 zinc finger proteins do not seem to have orthologs in *T. gondii*. The scarcity of conventional TFs presents a challenge to explain the sophisticated gene regulations in this parasite. In 2005, the discovery of APETALA2 family factors in apicomplexan parasites (ApiAP2) provided novel insights into the gene regulation puzzle.^[^ ^11^ ^]^ APETALA2 family transcription factors are widespread in plants, with important roles in regulating the metabolism, growth, environmental adaptation, and developmental programs of plants.^[^ ^12^ ^]^ ApiAP2 factors are greatly expanded in apicomplexan parasites and even more so in *T. gondii*, which has 67 in total. Whether these factors are bona fide transcription factors is to be determined, all these factors have one or more Apetala (AP2) DNA‐binding domain that contains ≈60 amino acid residues. Since their discovery, the functions of ApiAP2 have been extensively studied. The results show that they do have critical roles in regulating parasite development. For example, AP2‐G, AP2‐O, AP2‐SP in *Plasmodium spp* are key regulators of gametocytes, oocysts, and sporozoites development, respectively.^[^ ^13^ ^]^ These ApiAP2 factors may interact with additional ApiAP2 factors to achieve elaborated gene regulation networks.^[^ ^13^, ^14^ ^]^

Many ApiAP2 factors were also reported to regulate *T. gondii* cell cycle. The expression of more than one third of *T. gondii*’s AP2 proteins (24 out of 67) fluctuates during the cell cycle of tachyzoites.^[^ ^15^ ^]^ AP2X‐5, which has a peak expression during the S/M phases, interacts with AP2XI‐5 to regulate the cell‐cycle expression of a subset of micronemal and rhoptry proteins that contribute to parasite virulence.^[^ ^16^ ^]^ Deletion of AP2XII‐2, another S/M phase‐expressed factor, prolongs the S phase and increases the frequency of bradyzoite differentiation.^[^ ^17^ ^]^ Furthermore, AP2IX‐5, which is expressed exclusively in the early G1/S phase, is pivotal to regulate the expression of IMC (Inner Membrane Complex) and apical complex proteins that are key for daughter parasite formation during endopolygeny.^[^ ^18^ ^]^ In addition, a number of ApiAP2 factors were found to regulate bradyzoite differentiation and the establishment of chronic infection, by activating or repressing the expression of bradyzoite‐specific genes. AP2IV‐3, AP2Ib‐1, and AP2XI‐4 act as activators of bradyzoite differentiation, whereas AP2IX‐9, AP2IV‐4, and AP2IX‐4 function as inhibitors.^[^ ^19^ ^]^ How exactly these factors work together to regulate bradyzoite differentiation or reactivation is currently unknown. Their cooperations enable stage conventions timely and properly. Not surprisingly, ApiAP2 factors were also involved in the regulation of sexual development in *T. gondii*.^[^ ^20^ ^]^ MORC (microrchidia) and HDAC3 (Histone Deacetylase 3), two key epigenetic factors, form a complex to regulate sexual commitment.^[^ ^21^ ^]^ Their depletion leads to the upregulation of many merozoite and sexual stage specific genes. It is worth noting that the targets and functions of MORC and HDAC3 are quite diverse. In addition to the regulation of sexual commitment, they are also essential for tachyzoite growth. MORC and HDAC3 interact with different ApiAP2 factors, likely to specify the targets and regulate different biological processes. AP2XII‐1, which is predominantly expressed in tachyzoites, interacts with MORC and HDAC3 to inhibit the expression of merozoite specific genes, as well as to prevent merogony at the tachyzoite stage to enable rapid parasite proliferation by endodyogeny.^[^ ^20a,b^ ^]^

In this study, we found that the ApiAP2 factor AP2XII‐5 in *T. gondii*, forms a complex with MORC, HDAC3, and a novel nuclear protein called AIP1, to control the expression of a large set of developmentally regulated genes. In contrast to the inactivation of MORC or AP2XII‐1 that led to altered reproduction modes and subsequent growth arrest of tachyzoites, AP2XII‐5 and AIP1 were dispensable for tachyzoite growth, nor did they affect the reproduction modes of the parasites. These results suggest that the AP2XII‐5 containing complex has a more specific role of regulating gene expression during development.

## 2 Results

### 2.1 AP2XII‐5 is Dispensable for Tachyzoites Growth but is Needed for Optimal Proliferation under Bradyzoite‐Inducing Conditions In Vitro

**Figure 1:**
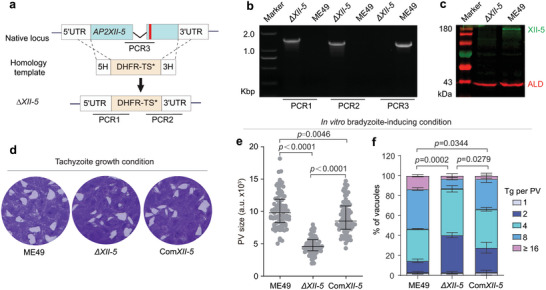
TgAP2XII‐5 is dispensable for tachyzoite growth in vitro. a) Schematic representation of knocking out *TgAP2XII‐5* by CRISPR/Cas9 assisted homologous gene replacement. Red bar indicates the CRISPR targeting site. b, c) Diagnostic PCRs and Western blotting on a representative clone of *ΔXII‐5*. d) Plaque assay comparing the overall growth of indicated strains under normal tachyzoite growth conditions. e, f) The parasitophorous vacuole (PV) size (e) and intracellular replication rates (f) of indicated strains under alkaline growth conditions (pH = 8.2, ambient CO_2_). (e) Median with interquartile range of more than 100 plaques, student's t‐test. (f) Means ± SEM of n = 3 independent experiments, each with three replicates, two‐way ANOVA with Tukey's multiple comparisons post‐tests.

AP2XII‐5 is a member of the AP2 family transcription factors possessing two AP2 DNA‐binding domains and an ACDC (AP2‐Coincident Domain mainly at the Carboxy‐terminus) domain at the C terminal region (Figure S1a, Supporting Information). To assess the function of *AP2XII‐5* in *T. gondii*, it was knocked out in the type II strain ME49 using CRISPR/Cas9‐mediated homologous recombination (**Figure** **1**a). Diagnostic PCRs demonstrated the replacement of *AP2XII‐5* by the drug selection marker DHFR* (Figure 1b). Western blotting using a homemade antibody against AP2XII‐5 further confirmed the deletion of the *AP2XII‐5* gene in the *ΔXII‐5* (ME49 *Δap2XII‐5*) mutant (Figure 1c). Prior to further characterization of the *ΔXII‐5* mutant, we constructed a complementing strain (Com*XII‐5*) by inserting an *AP2XII‐5* expressing cassette into the *HXGPRT* locus of the *ΔXII‐5* mutant (Figure S1b,c, Supporting Information). To assess the overall fitness of the transgenic strains, a plaque assay allowing the parasites to form plaques on monolayers of HFF cells was performed. The results show that the *ΔXII‐5* mutant grew equally well with the parental strain ME49 under standard tachyzoite growth conditions (Figure 1d). These results suggest that AP2XII‐5 is dispensable for tachyzoite growth in vitro.

A number of ApiAP2 factors have been shown to be involved in parasite development. To see whether AP2XII‐5 has such a role, we first checked the ability of the *ΔXII‐5* mutant to differentiate into bradyzoites in vitro, using an alkaline induction approach (pH = 8.2, ambient CO_2_). After growing the parasites in alkaline medium for three days, the bradyzoite development was probed by FITC conjugated DBA (Dolichos biflorus Agglutinin), which stained the wall of *Toxoplasma* cysts. The results showed that the *ΔXII‐5* mutant and ME49 had very similar bradyzoite transition rates (Figure S2, Supporting Information). On the other hand, in these bradyzoite induction experiments, we observed that the vacuoles formed by the *ΔXII‐5* mutant were smaller than those formed by ME49 or the complementing strain Com*XII‐5* (Figure 1e). To further quantify the growth differences between these strains under bradyzoite‐inducing conditions, we compared their intracellular replication rates. The results showed that the proliferation of the *ΔXII‐5* mutant was significantly slower than that of the wildtype strain and such defect was largely restored in the complementing strain (Figure 1f). Taken together, these results suggest that AP2XII‐5 is dispensable for the transition into bradyzoites in vitro but it is required for optimal parasite growth under bradyzoite‐inducing conditions.

### 2.2 AP2XII‐5 Suppresses the Expression of Development Activated Genes in Tachyzoites

**Figure 2:**
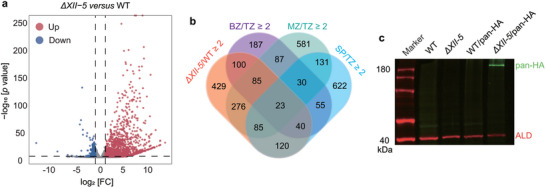
TgAP2XII‐5 inactivation leads to altered expression of a large number of developmentally regulated genes. a) Volcano plot showing differentially expressed genes in WT and *ΔXII‐5* strains, as determined by RNA‐seq. Data from three biological replicates were plotted. Red and blue dots indicate the number of genes that were significantly up‐ and down‐regulated (p values < 0.01 and fold change (FC) ≥ 2) in the *ΔXII‐5* mutant, respectively. b) Venn diagram illustrating the overlap between the genes silenced by AP2XII‐5 and those upregulated two‐fold or more in bradyzoites (BZ), merozoites (MZ), or sporozoites (SP) compared to tachyzoites (TZ) (data from ToxoDB). c) Western blot showing the protein expression of merozoite specific gene TgPAN in the WT and *ΔXII‐5* parasites. TgPAN was detected by an HA antibody in the ME49/pan‐HA or *ΔXII‐5*/pan‐HA transgenic strains.

The different impacts of *AP2XII‐5* deletion on parasite growth under tachyzoites and bradyzoites culture conditions implied that AP2XII‐5 might play a role during parasite development. To test this possibility, we first checked the gene expression changes caused by AP2XII‐5 deletion under both tachyzoite and bradyzoite growth conditions by RNA‐seq analyses. Using a fold change ≥2 and *p* value < 0.01 as parameters to identify differentially expressed genes, it was found that 1158 genes were upregulated and 193 were downregulated in the *ΔXII‐5* mutant grown under tachyzoite conditions (**Figure** **2**a). Similarly, AP2XII‐5 deletion caused increased transcript levels of 842 genes and decreased mRNA levels of 496 genes under bradyzoite growth conditions (Figure S3a, Supporting Information). The overall pattens of gene expression alteration under tachyzoite and bradyzoite conditions are very similar after *AP2XII‐5* deletion. For example, 649 of the 842 genes upregulated in *ΔXII‐5* under bradyzoite conditions were also upregulated in *ΔXII‐5* tachyzoites (Figure S3b, Supporting Information). To check the possible role of AP2XII‐5 on parasite development, we compared the gene expression changes caused by *AP2XII‐5* deletion with that during the life cycle of wildtype parasites. Such analyses revealed that the majority of genes affected by *AP2XII‐5* deletion were life cycle regulated. This is the most obvious for genes upregulated in the *ΔXII‐5* mutant since 63% of them are upregulated in wildtype bradyzoites, merozoites, or sporozoites compared to tachyzoites. More specifically, 21.4%, 40.5%, and 23.1% of the genes upregulated in the *ΔXII‐5* mutant grown under tachyzoite conditions are upregulated in wildtype bradyzoites, merozoites, and sporozoites respectively when compared to tachyzoites (Figure 2b). Similarly, 63.3% of the genes upregulated in the *ΔXII‐5* mutant cultured under bradyzoite‐inducing conditions are upregulated in wildtype bradyzoites, merozoites, or sporozoites (Figure S3c, Supporting Information). These results demonstrate that AP2XII‐5 suppresses the expression of development activated genes at the tachyzoite stage.

To further confirm the impact of AP2XII‐5 on the expression of developmentally regulated genes, an HA tag was inserted to the C‐terminus of the TGME49_235390 gene, which is PAN domain‐containing gene highly induced in the *ΔXII‐5* mutant. According to existing transcriptomic data, the expression of TGME49_235390 is extremely low in tachyzoites but is drastically increased in merozoites. After fusing the HA tag to the C‐terminus of the endogenous TGME49_235390 (PAN) gene in the ME49 and *ΔXII‐5* strains (pan‐HA) (Figure S4, Supporting Information), Western blotting (WB) probing the expression of HA was used to determine the protein levels of PAN. The results showed that pan‐HA could be easily detected in the *ΔXII‐5* mutant but not in the parental strain ME49 (Figure 2c), demonstrating that AP2XII‐5 deletion did increase the expression of developmentally regulated genes at both transcription and translation levels.

### 2.3 AP2XII‐5 Interacts with Novel Nuclear Factors to form a Protein Complex

**Figure 3:**
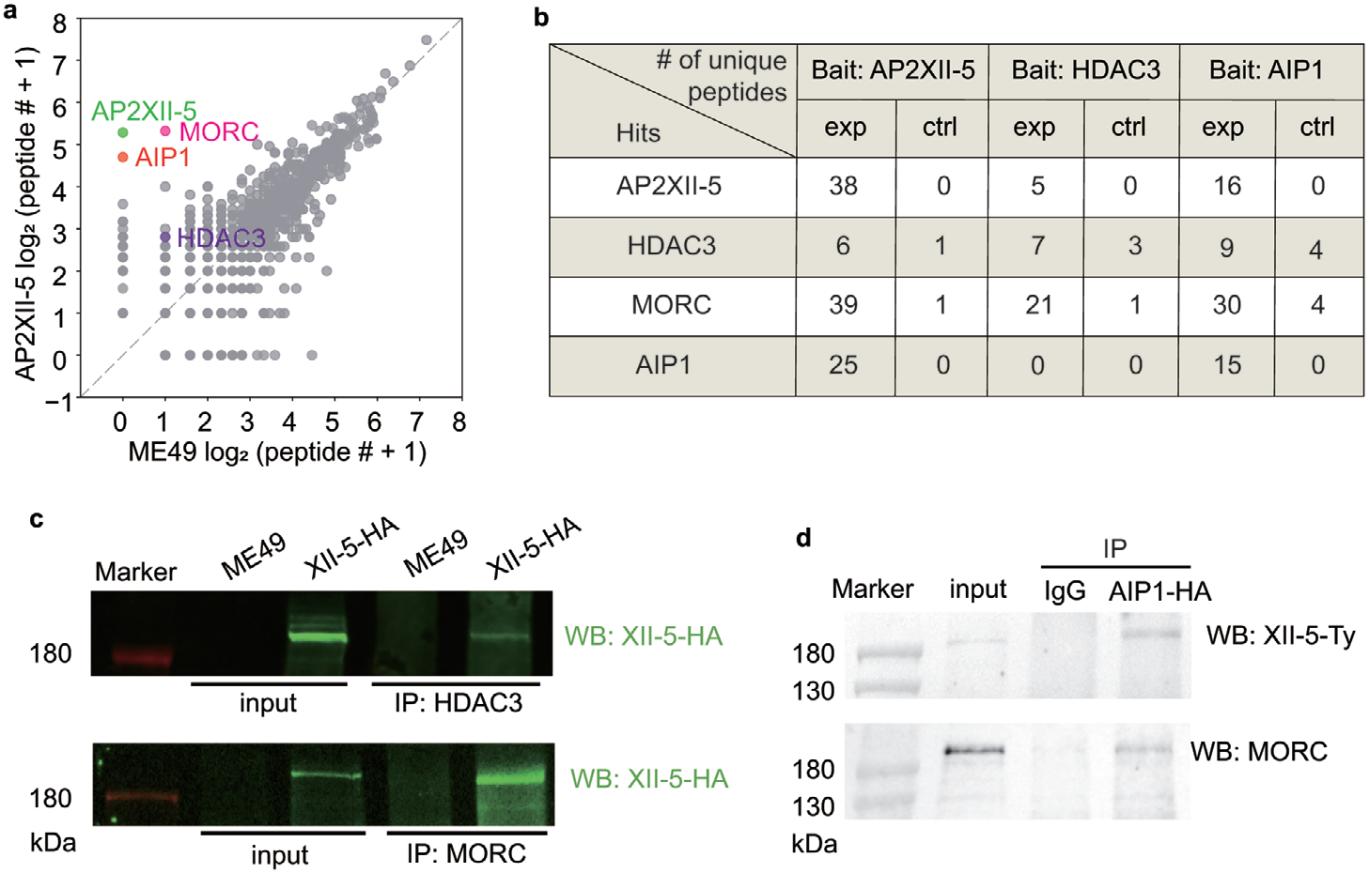
Identification of proteins interacting with TgAP2XII‐5. a) Proteins interacting with TgAP2XII‐5 were immunoprecipitated by an HA antibody from the lysate of the TgAP2XII‐5‐HA strain and identified by mass spectrometry. Co‐immunoprecipitation (co‐IP) using the ME49 strain was used as a control. The number of unique peptides for each identified protein in the co‐IP experiments was used to generate the plot. b) Selected proteins co‐precipitated with TgAP2XII‐5‐HA, HDAC3 or AIP1‐HA, as determined by mass spectrometry. The number of unique peptides for each hit in the experimental (exp) and control (ctrl, using untagged strains or negative serum for co‐IP) groups was indicated. A full list of hits was shown in Supplementary Data 1. c) Interactions between AP2XII‐5 and HDAC3 or MORC determined by co‐IP and Western blotting, using the TgAP2XII‐5‐HA strain. ME49 strain was used as a control. d) Interactions between AIP1 and AP2XII‐5‐Ty or MORC determined by co‐IP and Western blotting, using the AIP1‐mAID/XII‐5‐Ty strain.

To figure out the mechanisms by which AP2XII‐5 regulates the expression of target genes, we first sought to identify the proteins that interact with it. For this purpose, we used the AP2XII‐5‐HA strain in a co‐immunoprecipitation (co‐IP) experiment to pull down the proteins interacting with AP2XII‐5 using an HA monoclonal antibody. Subsequently the precipitated proteins were identified by mass spectrometry (MS). AP2XII‐5 was among the top 2 hits from the co‐IP/MS experiments using the AP2XII‐5‐HA strain. In contrast, no AP2XII‐5 peptides were detected in the control group using the parental strain ME49 (**Figure** **3**a), demonstrating the specificity of the experiment. Using this approach, a number of proteins were found to be co‐precipitated with AP2XII‐5‐HA, including MORC and HDAC3 that were shown to program gene expression during parasite development (Figure 3a,b). In addition, we also found a previously uncharacterized protein (TGME49_314220) to be obviously enriched in the AP2XII‐5‐HA‐based co‐IP/MS (Figure 3a,b). The function of this protein is completely unknown and BLAST searches only identified homologs in parasites that are closely related to *T. gondii*, such as *Hammondia hammondi* and *Neospora caninum*. As such, we named this protein as AIP1 (AP2XII‐5 interacting protein 1) because of its interaction with AP2XII‐5 (see below). To further confirm that these proteins formed a complex, we performed additional co‐IP experiments using HDAC3 and AIP1 as baits. The results showed that AP2XII‐5, MORC, HDAC3, and AIP1 could be reciprocally precipitated, except that AIP1 was not present in HDAC3 based co‐IP/MS (Figure 3b). Protein interactions in this complex were also examined by co‐IP and subsequent Western blotting (WB). The results showed that AP2XII‐5 was successfully co‐precipitated with MORC or HDAC3 in the AP2XII‐5‐HA strain (Figure 3c), which contained a spaghetti monster HA tag fused to the C‐terminus of endogenous AP2XII‐5 (Figure S5, Supporting Information). These results confirmed the interactions between AP2XII‐5 and MORC, as well as HDAC3. Similarly, using the AIP1‐mAID/XII‐5‐Ty strain (described below), which contained an mAID degron (HA tagged) fused to the C‐terminus of endogenous AIP1 and expressed a second copy of AP2XII‐5 (Ty tagged) from the *HXGPRT* locus, AP2XII‐5 and MORC were also found to be co‐precipitated with AIP1 (Figure 3d). Taken together, these data demonstrated that AP2XII‐5 was in a protein complex that contained MORC, HDAC3, and AIP1 in *Toxoplasma* tachyzoites.

### 2.4 AIP1 Deletion Leads to Similar Gene Expression Changes as AP2XII‐5 Inactivation

In addition to AP2XII‐5, MORC, and HDAC3 in the AP2XII‐5‐containing complex were also shown to regulate the expression of developmentally regulated genes.^[^ ^21b^ ^]^ This prompted us to assess the physiological roles of AIP1 in the parasites. For this purpose, a conditional AIP1 depletion strain AIP1‐mAID (iAIP1) was constructed by adding a mAID degron (HA tagged) to the carboxyl terminus of endogenous AIP1 in the ME49 Tir1 strain (Figure S6a, Supporting Information). Both IFA and Western blotting confirmed the degradation of AIP1‐mAID fusion in an IAA (Indole‐3‐acetic acid) dependent manner (Figure S6b,c, Supporting Information). In addition, IFA also confirmed the localization of AIP1 to parasite nucleus, which is consistent with its interaction with AP2XII‐5 that resides in the parasite nucleus (Figure S6c, Supporting Information). Similar to the *ΔXII‐5* mutant, depletion of AIP1 did not have a noticeable impact on parasite replication or growth under tachyzoite growth conditions in vitro (Figure S7a,b, Supporting Information). In contrast, under alkaline growth conditions that induced bradyzoite transition, the AIP1 depleted mutant had a bradyzoite transition rate close to 100%, much higher than that of the AIP1 expressing strains, which were below 40% (for an unknown reason, the bradyzoite transition rate of the ME49 Tir1 strain was much lower than its parental strain ME49) (Figure S7c, Supporting Information). Along with the increased bradyzoite transition rate, the intracellular replication rate of the AIP1 depletion mutant was also slightly reduced under alkaline conditions (Figure S7d, Supporting Information).

**Figure 4:**
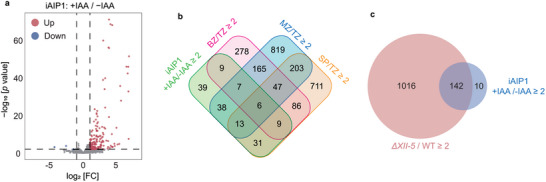
TgAIP1 suppresses the expression of developmentally activated genes in tachyzoites. a) Volcano plot of differentially expressed genes before (‐IAA) and after (+IAA, 24 hours) AIP1 depletion in the AIP1‐mAID (iAIP1) strain, as determined by RNA‐seq analyses. Data from three biological replicates were plotted. Genes significantly up‐ or down‐regulated were defined as *p* < 0.01 and fold change (FC) ≥ 2. b) Venn diagram illustrating the overlap between genes silenced by TgAIP1 and the genes that are upregulated at different life cycle stages compared to tachyzoites (Source: ToxoDB), as done in Figure 2b. c) Venn diagram illustrating the overlap of genes suppressed by TgAIP1 and TgAP2XII‐5.

To assess the gene expression changes caused by AIP1 depletion, transcriptomic analyses using RNA‐Seq were applied to the iAIP1 tachyzoites treated with or without IAA for 24 h. A total of 155 differentially expressed genes were identified, including 3 downregulated genes and 152 upregulated genes after AIP1 depletion (**Figure** **4**a). Interestingly, the majority of the genes upregulated in the AIP1 depleted mutants were developmentally regulated in wildtype parasites. For example, 42.1%, 38.8%, and 20.4% of them were the genes activated at the merozoite, sporozoite, and bradyzoite stages, respectively (Figure 4b). This pattern of gene expression change is very similar to that caused by AP2XII‐5 deletion. Of the 152 genes upregulated in the AIP1 depletion mutant, 142 were also upregulated in the *ΔXII‐5* mutant (Figure 4c). These results are consistent with the above findings showing that AP2XII‐5 and AIP1 interact with each other to form a complex, which tunes the expression of developmentally regulated genes.

### 2.5 AIP1 Stabilizes the AP2XII‐5‐Containing Complex to Suppress the Expression of Development Activated Genes in Tachyzoites

**Figure 5:**
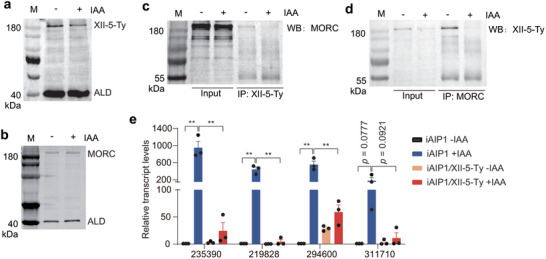
TgAIP1 stabilizes the AP2XII‐5‐MORC complex to suppress the expression of target genes. a, b) Protein levels of AP2XII‐5 and MORC in the iAIP1/XII‐5‐Ty strain treated with or without IAA. c, d) Protein interactions between AP2XII‐5 and MORC in the iAIP1/XII‐5‐Ty strain with or without IAA treatment, as determined by coIP and Western blotting. M: protein marker. e) Expression changes of selected genes in the indicated strains treated with or without IAA, as determined by RT‐qPCR. TgME49_235390 and TgME49_219828 are merozoite specific genes, while TgME49_294600 and TgME49_311710 are sporozoite specific genes. Beta tubulin was used as a normalization control. Means ± SEM of n = 3 independent experiments, unpaired two‐tailed student's t‐test. **P < 0.01.

To further elucidate the mode of function of AIP1, a minigene expressing Ty tagged AP2XII‐5 driven by the GRA1 promoter was inserted into the *HXGPRT* locus of iAIP1 to generate the iAIP1/XII‐5‐Ty strain (Figure S8, Supporting Information), which over‐expressed AP2XII‐5 because it had two copies of AP2XII‐5 (the endogenous one and pGRA1::AP2XII‐5 expressed from the *HXGPRT* locus). First, we estimated the protein abundance of AP2XII‐5 and MORC before and after AIP1 depletion. Western blotting analyses on the iAIP1/XII‐5‐Ty strain found that the protein level of AP2XII‐5 was slightly reduced after AIP1 depletion, but that of MORC was not affected (**Figure** **5**a,b). We also tested the stability of the AP2XII‐5‐containing complex by co‐IP and WB. The results showed that the amount of AP2XII‐5 co‐precipitated with MORC or the amount of MORC co‐precipitated with AP2XII‐5 was noticeably reduced after AIP1 depletion, likely because of the reduced levels of AP2XII‐5 and/or decreased interactions between AP2XII‐5 and MORC in the absence of AIP1 (Figure 5c,d). These data suggest that AIP1 has a role to stabilize the AP2XII‐5‐containing complex.

The reduced level of AP2XII‐5 and decreased stability of the AP2XII‐5‐containing complex after AIP1 depletion prompted us to investigate whether increasing the expression of AP2XII‐5 could restore the gene expression changes caused by AIP1 depletion. To this end, the iAIP1 and iAIP1/XII‐5‐Ty (which overexpressed AP2XII‐5 in iAIP1, as explained above) strains treated with or without IAA were subjected to quantitative reverse transcription polymerase chain reaction (RT‐qPCR) analyses, to examine the transcript levels of selected developmentally regulated genes (Figure 5e). Consistent with the RNA‐Seq results, the mRNA levels of TgME49_235390, TgME49_219828, TgME49_294600, and TgME49_311710 in the AIP1 depleted parasites were drastically increased (comparing the iAIP1 +IAA versus iAIP1 ‐IAA groups) (Figure 5e). Interestingly, AP2XII‐5 overexpression significantly prevented the increase of mRNA levels of these genes in AIP1 depleted parasites (comparing the iAIP1 +IAA versus iAIP1/XII‐5‐Ty +IAA groups). These results support our hypothesis and suggest that AIP1 stabilizes the AP2XII‐5‐containing complex to regulate the expression of target genes.

### 2.6 AP2XII‐5 Recruits HDAC3 to Inhibit the Expression of Target Genes

**Figure 6:**
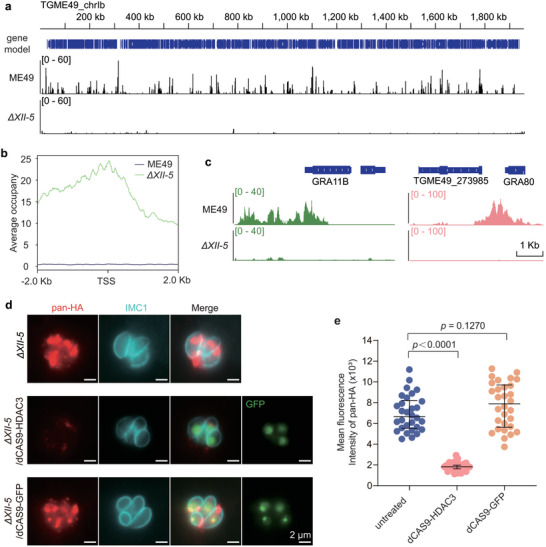
AP2XII‐5 recruits HDAC3 to repress the expression of target genes. a) Binding sites of HDAC3 on the genomes of ME49 and the ΔXII‐5 strains, as determined by ChIP‐Seq. Chromosome Ib was shown as an example. The y‐axis depicts the abundance of ChIP‐Seq reads. b) AP2XII‐5 dependent recruitment of HDAC3 to the upstream regions of genes whose transcription was upregulated after *AP2XII‐5* deletion. Binding of HDAC3 to the ‐2 kb to +2 kb regions of the transcription start sites (TSS) of target genes in the ME49 and the ΔXII‐5 strain was plotted. Data from n = 2 replicates of each strain were included. c) Binding of HDAC3 to the promoter regions of representative merozoite specific genes (GRA11B, TGME49_273985, and TgGRA80) in an AP2XII‐5 dependent manner, as determined by ChIP‐Seq. d‐e) Artificial delivery of HDAC3 to the promoter region of *PAN* in the *ΔXII‐5* strain reduced *PAN* expression. HDAC3 was fused to dCas9‐GFP and expressed from a plasmid that also expressed a *PAN* targeting sgRNA (sgPAN). The plasmid (or the dCas9‐GFP empty vector as a negative control) was introduced into the ΔXII‐5 strain and IFA analysis was performed using an anti‐HA antibody to probe the expression of pan‐HA. Representative images taken with the same exposure time to compare the fluorescence intensity of pan‐HA were shown in (d). Quantification of the fluorescence intensity of pan‐HA in n ≥ 30 transfectants (indicated by GFP^+^ in the nuclei) was plotted in (e). Median with interquartile range of three independent experiments, unpaired two‐tailed Student's t‐test.

AP2XII‐5 forms a complex with MORC and HDAC3, which erases histone acetylation and reduces chromatin accessibility to silence the expression of genes. To see if the inhibition of target gene expression by AP2XII‐5 depends on HDAC3 or not, we performed chromatin immunoprecipitation followed by DNA sequencing (ChIP‐Seq) to determine the HDAC3 binding sites in the genomes of ME49 and *ΔXII‐5* strains using CUT&Tag. Our results show that HDAC3 widely occupied the genome of ME49, mostly at intergenic regions. In contrast, chromosomal occupation of HDAC3 was dramatically reduced in the *ΔXII‐5* mutant (**Figure** **6**a). A closer look at HDAC3 binding sites in ME49 versus *ΔXII‐5* revealed that many of the genes with increased mRNA levels in the *ΔXII‐5* mutant were targeted by HDAC3 at their promoter regions in ME49 (Figure 6b). But *AP2XII‐5* deletion abolished HDAC3 binding to these sites (Figure 6b). For example, HDAC3 was enriched in the promoter regions of the merozoite specific genes GRA11B and GRA80 in ME49 but not in the *ΔXII‐5* mutant (Figure 6c). Given the gene silencing function of HDAC3, these results indicate that AP2XII‐5 probably recruits HDAC3 to the promoter regions of target genes to suppress their expression.

To further confirm that AP2XII‐5 depends on HDAC3 to inhibit target gene expression, we artificially delivered HDAC3 to the promoter regions of target genes in *ΔXII‐5* using an AP2XII‐5‐independent approach, and then checked their transcription levels. For this purpose, we fused HADC3 to an enzymatic dead mutant of Cas9 called dCAS9 (contained the D10A and H840A mutations that abolished its nuclease activity), which was then directed to the promoter region of the merozoite‐specific gene *PAN* by a target specific sgRNA. Consistent with the Western blotting results shown in Figure 2c, IFA analyses demonstrated that PAN‐HA could be readily detected in the *ΔXII‐5* mutant (Figure 6d). In contrast, when dCAS9‐HDAC3/sgRNA was delivered to the *ΔXII‐5/*pan‐HA strain, PAN‐HA was barely detected and the signal intensity was significantly lower (reduced by ≈70%) than that in the absence of dCAS9‐HDAC3 (Figure 6d,e). As a control, the dCAS9‐GFP fusion did not have such an effect (Figure 6d,e). These results suggest that when delivered to the right genomic region, HDAC3 could suppress the expression of target gene in the absence of AP2XII‐5, further supporting that AP2XII‐5 inhibited the expression of target genes by recruiting HDAC3 in wildtype strains.

### 2.7 Disruption of AP2XII‐5 Reduced Parasite Virulence In Vivo

**Figure 7:**
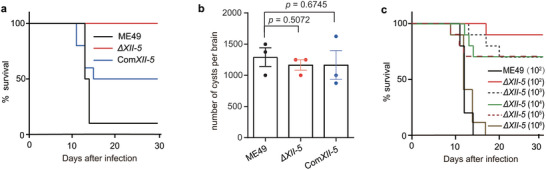
AP2XII‐5 is crucial for parasite virulence in vivo. a) Survival curves of KM mice infected with different strains. The infection dose is 100 parasites per mouse and each strain was tested with 10 mice. b) Number of cysts in the brains of mice that survived in (a). Means ± SEM of n = 3 mice from each group were analyzed, unpaired two‐tailed Student's t‐test. c) Dose dependent virulence of the *ΔXII‐5* mutant. KM mice were injected with indicated doses of ME49 versus *ΔXII‐5* tachyzoites and the survival of mice was monitored for 30 days. Each dose was tested by n = 10 mice.

The results described above show that AP2XII‐5 is dispensable for tachyzoite growth in vitro. To check if it is needed for parasite virulence in vivo, purified tachyzoites of the *ΔXII‐5* mutant were used to infect KM mice through peritoneal injection. Subsequently, the symptoms and survival of infected animals were monitored for 30 days. At a low infection dose (100 parasites per mouse), the parental strain ME49 caused 90% mortality in mice. In contrast, the *ΔXII‐5* mutant did not cause any animal death. AP2XII‐5 complementation partially restored the virulence as the complementing strain Com*XII‐5* caused 50% mortality in mice, suggesting that AP2XII‐5 contributed to the acute virulence of the parasites (**Figure** **7**a). We also checked the impact of *AP2XII‐5* deletion on the establishment of chronic infections. Thirty days after infection, surviving mice were euthanized and the number of cysts in the brains was determined by IFA using fluorescein isothiocyanate conjugated Dolichos biflorus agglutinin, which stained the cyst wall. Interestingly, although *ΔXII‐5* mutant displayed attenuated virulence, its capacity to form cyst in vivo was indistinguishable from that of the ME49 or Com*XII‐5* strains (Figure 7b). To further examine the degree of virulence attenuation of the *ΔXII‐5* mutant, different doses of the *ΔXII‐5* mutant were used to infect mice. At the infection doses of 10^2^ to 10^5^ parasites per mouse, mice infected with the *ΔXII‐5* mutant had survival rates of 70% or higher. At the dose of 10^6^ parasites per mouse, infection with the *ΔXII‐5* mutant resulted in 100% death (Figure 7c). Given that the parental strain ME49 caused 100% mortality at the infection dose of 100 parasites per mouse, these results suggest that the *ΔXII‐5* mutant was attenuated ≈10^4^ fold.

### 2.8 Reduced Protein Levels of AP2XII‐5 During the Transition into Merozoites

**Figure 8:**
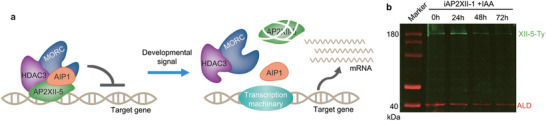
A model for the role of the AP2XII‐5 containing complex in controlling the expression of developmentally regulated genes. a) AP2XII‐5 forms a complex with AIP1 and the epigenetic factors MORC and HDAC3 to silence the expression of target genes at the tachyzoite stage. When the parasites are stimulated to differentiate, the abundance of the AP2XII‐5 protein was decreased by an unknown mechanism, so that the repression of target genes was relieved. b) Western blotting checking the abundance of TgAP2XII‐5 in the iAP2XII‐1/AP2XII‐5‐Ty strain treated with IAA for different periods (0–72 h) that induced merogony.

The above results indicate that AP2XII‐5 forms a complex with AIP1, MORC, and HDAC3 to suppress the expression of development activated genes at the tachyzoite stage (**Figure** **8**a). On the other hand, the inhibitory effect of AP2XII‐5 needs to be relieved in wildtype parasites when the parasites do differentiate into other stages like merozoites, so that the development programs can proceed. But how the inhibitory effect of AP2XII‐5 is relieved is not known. We and others have previously shown that AP2XII‐1 depletion leads to a merozoite‐like state of the parasites in vitro.^[^ ^20a,b^ ^]^ As such, we compared the protein abundance of AP2XII‐5 before and after AP2XII‐1 depletion. For this purpose, we constructed an iAP2XII‐1/XII‐5‐Ty strain by inserting a Ty tag to the C‐terminus of endogenous AP2XII‐5 in the iAP2XII‐1 strain we generated previously (Figure S9a,b, Supporting Information).^[^ ^20a^ ^]^ Then, the iAP2XII‐1/XII‐5‐Ty strain was treated with IAA to deplete AP2XII‐1 (Figure S9c, Supporting Information), and the protein levels of AP2XII‐5 was determined by Western blotting using a Ty antibody. The results showed that the level of AP2XII‐5 was gradually reduced as the induction of AP2XII‐1 depletion prolonged (Figure 8b). Existing transcriptomic data imply that the mRNA level of AP2XII‐5 does not fluctuate much at different developmental stages (data from ToxoDB), suggesting that the protein abundance of AP2XII‐5 is probably regulated post‐transcriptionally. Together, these results indicate that the AP2XII‐5 protein is degraded (or reduced by other means) or translationally inhibited when the parasites differentiate into merozoites, thus relieving its inhibitory effect on the expression of merozoite specific genes and merozoite development can proceed.

## 3 Discussion

The developmental programs in the life cycle of *T. gondii* are precisely regulated, so that stage transitions do occur and only occur when necessary. Although factors like BFD1 that serves as a master regulator of bradyzoite formation have been identified, the regulatory mechanisms underlying the complex life cycle of *T. gondii* is largely unknown.^[^ ^22^ ^]^ Previously, a number of factors, including ApiAP2 family transcription factors and epigenetic factors like MORC and HDAC3, were shown to regulate parasite development.^[^ ^17^, ^19^, ^20^, ^21^ ^]^ But how exactly these factors coordinate to control the life cycle progression remains to be elucidated. In this study, we identified the ApiAP2 factor AP2XII‐5 as a critical regulator for the expression of developmentally regulated genes. AP2XII‐5 forms a complex with MORC and HDAC3, as well as a novel nuclear factor AIP1 to suppress the expression of developmentally activated genes at the tachyzoite stage. Although AP2XII‐5 interacts with MORC and HDAC3, two histone modifiers with key roles in regulating parasite development, disruption of AP2XII‐5 did not have the same cellular phenotypes as the inactivation of MORC or HDAC3. Our results suggest a model that, AP2XII‐5 as a DNA binding factor, binds and specifies the targets to be regulated, and exerts its gene‐repressing function by recruiting the epigenetic modifiers MORC and HDAC3 to target sites. However, AP2XII‐5 has a narrower range of targets than MORC and HDAC3, and it is only involved in gene expression regulation during parasite development.

Transition from one developmental stage to another involves many changes in the parasites, such as metabolic activities, reproduction modes, gene expression patters and morphogenesis. Previously, several factors, including MORC, HDAC3, AP2XII‐1 and AP2XI‐2, were shown to suppress the expression of merozoite or sexual stage specific genes in tachyzoite,^[^ ^20^, ^21^ ^]^ similar to AP2XII‐5. However, inactivation of those genes had more dramatic cellular consequences than deletion of *AP2XII‐5*. Suppressing the functions of MORC, HDAC3, AP2XII‐1, or AP2XI‐2 by genetic or chemical genetic means all changed the reproduction mode of parasites from endodyogeny to merogony, and induced commitment to sexual development. Meanwhile, these four genes are also critical for tachyzoite proliferation.^[^ ^20^, ^21^ ^]^ These results suggest that MORC, HDAC3, AP2XII‐1, and AP2XI‐2 have multiple functions in regulating parasite growth and development. In contrast, AP2XII‐5 is dispensable in tachyzoites and loss of AP2XII‐5 did not change the reproduction mode of the parasites (Figure S10, Supporting Information). AP2XII‐5 specifically controls the expression of a set of developmentally regulated genes. Interestingly, the inhibition of developmentally activated genes in tachyzoites by AP2XII‐5 depends on HDAC3. Deletion of AP2XII‐5 abolished the recruitment of HDAC3 to target genes on the chromosome. In addition, artificial delivery of HDAC3 to the promoter region of target gene re‐suppressed its expression in the AP2XII‐5 deletion mutant. Together, these results suggest that, by interacting with MORC and HDAC3, AP2XII‐5 only affects the expression of a subset of MORC and HDAC3's targets. Therefore, the phenotypes of AP2XII‐5 deletion and MORC or HDAC3 inactivation only partially overlap. The impact of AP2XII‐5 deletion is more specific, concentrating on the expression control of developmentally regulated genes. Consistent with this hypothesis, MORC was previously found to interact with a number of so‐called primary ApiAP2 factor,^[^ ^21b^ ^]^ which likely instruct MORC and HDAC3 to regulate different subsets of genes that are involved in different aspects of parasite activities. Interestingly, AP2XII‐5 is one of those primary ApiAP2 factors.^[^ ^21b^ ^]^

Due to ethical issues of animal experiments involving cats, the study of *Toxoplasma* sexual development is fairly challenging. The vast majority of limited work done so far was performed in vitro. While the depletion of MORC or AP2XII‐1 was shown to induce sexual commitment and merozoite formation, how different these induced merozoites are from the wildtype merozoites formed in cats is unknown. But it is clear that those induced merozoites did not survive long in tissue culture and rarely proceeded further to produce gametes or oocysts.^[^ ^20^, ^21^ ^]^ It is possible that, by manipulating these factors, the changes are too dramatic and the parasites died before further development. As such, more sophisticated manipulations that only channel the parasite development to sexual differentiation will be needed. Deletion of AP2XII‐5 could be one of the steps since it causes merozoite‐like gene expression patterns. Manipulating additional factors that tune the parasite activities like merogony may be combined with AP2XII‐5 deletion to achieve viable and bioactive merozoites in vitro.

Also due to the ethical and technical difficulties, how the activities of known factors involved in parasite development, like MORC, HDAC3, and AP2XII‐1, are regulated is not known. For MORC and HDAC3 that suppress merozoite differentiation in tachyzoites, their inhibitory effects need to be removed during bona fide merozoite development. But the mechanism is currently unknown. Existing transcriptomic data suggest that although their transcription seems to be low in un‐sporulated and sporulating oocysts, they are expressed in both tachyzoite and merozoite stages, suggesting that other means may regulate the activities of MORC and HDAC3. One possibility is that they interact with different factors like ApiAP2 at different stages, which allows the regulation of different targets by MORC and HDAC3. For AP2XII‐1, it does not seem to be transcribed robustly in merozoites and seems to be a tachyzoite specific gene.^[^ ^20a^ ^]^ Therefore, the activity of AP2XII‐1 may be restricted in tachyzoites by transcriptional control mechanisms. AP2XII‐5, which inhibits the expression of many bradyzoite, merozoite and sporozoite specific genes in tachyzoites, seems to be actively transcribed across all life stages (Data in ToxoDB). Using the AP2XII‐1 depletion strain that induced merozoite transition, we showed that the protein levels of AP2XII‐5 were gradually reduced when merozoite induction proceeded. These results suggest that, likely by means like translational control, posttranslational modification, and protein degradation, the activity of AP2XII‐5 is reduced in merozoites and its inhibitory effects on merozoite gene expression is relieved during merozoite development. But how exactly the abundance and activity of AP2XII‐5 is regulated during the parasite life cycle is still an open question, which deserves further investigations.

## 4 Experimental Section

### Parasite Strains and Cell Cultures

Type II strains, including ME49, ME49 *Δhxgprt*::Tir1 (ME49 Tir1), and the transgenic strains derived from them, were cultured in human foreskin fibroblasts (HFF) (ATCC, USA) as previously described.^[^ ^23^ ^]^ HFF cells were maintained in Dulbecco's Modified Eagle Medium (DMEM) (Sigma‐Aldrich, USA), supplemented with heat‐inactivated fetal bovine serum (FBS) (10%) (Gibco, USA), penicillin‐streptomycin (1%), andL‐glutamine (5 mM). To induce bradyzoite differentiation in vitro, parasites were cultured in RPMI 1640 medium (pH = 8.2) containing 1% fetal bovine serum, along with ambient CO_2_.

### Construction of Recombinant Plasmids

All strains used in this study are listed in Table S1 (Supporting Information). All plasmids and primers used are listed in Tables S2 and S3 (Supporting Information) respectively. Locus‐specific CRISPR plasmids were generated by replacing the UPRT‐targeting sgRNA sequence in pSAG1::Cas9‐U6::sg*UPRT* with locus‐specific guide sequences, following established protocols.^[^ ^24^ ^]^ Other plasmids were constructed using multi‐fragment cloning with the ClonExpress MultiS Cloning Kit (Vazyme Biotech, China). The pUC19‐AP2XII‐5‐DHFR plasmid was constructed by cloning the 5′ and 3′ homologous arms of AP2XII‐5 amplified from ME49 genomic DNA, and the DHFR* fragment amplified from pDONR‐DHFR into the pUC19 vector. Similarly, pUC19‐AP2XII‐5‐HA‐DHFR, pUC19‐AP2XII‐5‐Ty‐DHFR, and pUC19‐PAN‐HA‐CAT plasmids were constructed using the same approach. For the construction of these plasmids, the HA‐DHFR fragment was amplified from pUC19‐smHA‐3′UTR‐dhfr, the Ty‐DHFR fragment was amplified from pUC19‐Ty‐3′UTR‐DHFR, and the HA‐CAT fragment was from pUC19‐smHA‐3′UTR‐CAT. The pUC19‐AP2XII‐5‐COMP plasmid was constructed by assembling the tubulin promoter, AP2XII‐5 coding sequence, and the HA‐CAT fragment into the pUC19 vector. The AP2XII‐5 coding sequence was amplified from cDNA prepared from the ME49 tachyzoites. The tubulin promoter and HA‐CAT fragments were amplified from pUC19‐pTub:AP2X‐4‐HA∼CAT. Similarly, the pUC19‐pGRA1‐ap2XII‐5‐Ty plasmid was constructed in a similar way like pUC19‐AP2XII‐5‐COMP. The GRA1 promoter was amplified from ME49 genomic DNA and the Ty‐DHFR fragment was amplified from pUC19‐Ty‐3′UTR‐DHFR. The pUC19‐AIP1‐mAID‐HXGPRT plasmid was generated by cloning the 5′ and 3′ homologous arms of AIP1 and the mAID‐HXGPRT fragments into pUC19. The homologous arms were amplified from ME49 genomic DNA, while the mAID‐HXGPRT fragment was amplified from pTUB1:YFP‐mAID‐3HA, DHFR‐TS:HXGPRT. pSRS34A::dCAS9‐EGFP‐KRAB‐U6::sg*UPRT* was constructed by assembling the SRS34 promoter, KRAB coding sequence, and the dCas9‐U6::sg*UPRT* fragment. Specifically, the SRS34 promoter was amplified from the RH genome, the KRAB was amplified from pHR‐SFFV‐dCas9‐BFP‐KRAB, and the dCas9‐U6::sg*UPRT* fragment was amplified from pSAG1::dCas9‐U6::sg*UPRT*. The pSRS34A::dCAS9‐EGFP‐HDAC3‐U6::sg*PAN* plasmid was constructed in two steps. First, the HDAC3 coding sequence was amplified from ME49 cDNA and used to replace the KRAB sequence in the pSRS34A::dCAS9‐EGFP‐KRAB‐U6::sg*UPRT* plasmid, generating pSRS34A::dCAS9‐EGFP‐HDAC3‐U6::sg*UPRT*. Then, the UPRT‐targeting sgRNA sequence in pSRS34A::dCAS9‐EGFP‐HDAC3‐U6::sg*UPRT* was replaced with the PAN‐targeting sgRNA. The pSRS34A::dCAS9‐EGFP‐U6::sg*PAN* plasmid was constructed by deleting HDAC3 in pSRS34A::dCAS9‐EGFP‐HDAC3‐U6::sg*PAN*, using the ClonExpress II One Step Cloning Kit (Vazyme Biotech, China).

### Construction of Transgenic Strains

All transgenic strains were generated via CRISPR/Cas9‐mediated site‐specific gene editing. The *ΔXII‐5* strain was constructed by replacing the AP2XII‐5 gene with the DHFR* selection marker. The AP2XII‐5‐DHFR fragment amplified from pUC19‐AP2XII‐5‐DHFR was co‐transfected into freshly egressed tachyzoites of ME49 with the pSAG1::Cas9‐U6::sg*AP2XII‐5* plasmid. The transfectants were then selected with pyrimethamine (1 µM) and single cloned by limiting dilution in 96 well plates. The AP2XII‐5‐HA strain was created by co‐transfecting the pSAG1::Cas9‐U6::sg*AP2XII‐5*‐loc plasmid and the AP2XII‐5‐HA‐DHFR fragment (derived from the pUC19‐AP2XII‐5‐HA‐DHFR plasmid) into ME49, with pyrimethamine selection as above. Similarly, the ME49/pan‐HA and *ΔXII‐5*/pan‐HA strains were constructed by transfecting pSAG1::Cas9‐U6::sg*PAN*‐loc plasmid and PAN‐HA‐CAT fragment (derived from the pUC19‐PAN‐HA‐CAT plasmid) into fresh tachyzoites of ME49 or *ΔXII‐5*, selected with chloramphenicol (30 µM), and single cloned by limiting dilution. The APXII‐5 complementation strain (Com*XII‐5*) was created by co‐electroporating AP2XII‐5 expression cassettes (amplified from pUC19‐AP2XII‐5‐COMP) and the pSAG1::Cas9‐U6::sg*HXGPRT*‐intron plasmid into the *ΔXII‐5* strain, followed by selection with chloramphenicol (30 µM). The AIP1‐mAID strain was constructed by transfecting pSAG1::Cas9‐U6::sg*AIP1* plasmid and AIP1‐mAID‐HXGPRT fragment (derived from the pUC19‐AIP1‐mAID‐HXGPRT plasmid) into fresh tachyzoites of ME49 Tir1, and selected with mycophenolic acid (25 µg mL^−1^, MPA) and xanthine (50 µg mL^−1^, Sigma‐Aldrich, USA). The iAIP1/XII‐5‐Ty strain was constructed by co‐electroporating the AP2XII‐5 expression cassette (amplified from pUC19‐pGRA1‐ap2XII‐5‐Ty) and the pSAG1::Cas9‐U6::sg*HXGPRT*‐intron plasmid into AIP1‐mAID strain, followed by selection with pyrimethamine (1 µM). The iAP2XII‐1/XII‐5‐Ty strain was constructed by co‐transfecting pSAG1::Cas9‐U6::sg*AP2XII‐5*‐loc and the AP2XII‐5‐Ty‐DHFR fragment (amplified from pUC19‐AP2XII‐5‐Ty‐DHFR) into the AP2XII‐1‐mAID strain, followed by selection with pyrimethamine (1 µM). In all cases, single clones were verified by diagnostic PCR and/or immunofluorescence assays (IFA) before use.

### Immunofluorescent Assays (IFA) and Western Blots (WB)

All IFAs and WBs to monitor protein expression were performed following established protocols.^[^ ^24^, ^25^ ^]^ The primary antibodies used in this study included: rabbit anti‐TgALD (1:1000 for IFA; 1:8000 for WB), mouse anti‐Ty (BB2 clone of anti‐Ty1, provided by Prof. David Sibley, Washington University in St. Louis, USA) (1:1000 for IFA; 1:4000 for WB), mouse anti‐HA (TANA2 clone of anti‐HA, MBL International Corporation, Japan) (1:1000 for IFA; 1:5000 for WB), rabbit anti‐TgIMC1 (generated by immunizing rabbit with AA 172–342 of recombinant *T. gondii* IMC1) (1:2000 for IFA), mouse anti‐TgGAP45 (provided by Dr. Jinlei Wang, Lanzhou Veterinary Research Institute, China) (1:500 for IFA), mouse anti‐TgAP2XII‐5 (generated by immunizing mouse with AA 407–707 of recombinant *T. gondii* AP2XII‐5 as antigen, 1:200 for WB), mouse anti‐TgMORC (generated by immunizing mouse with AA 61–452 of recombinant *T. gondii* MORC as antigen, 1:4000 for WB), and rabbit anti‐TgHDAC3 (generated by immunizing rabbit with the synthetic peptide sequence “CPYRIRRKDYANDFE” conjugated to KLH, 1:2000 for WB). The secondary antibodies included: IRDye 680RD Goat anti‐Rabbit IgG (1:5000 for WB), IRDye 800CW Goat anti‐Mouse IgG (1:5000 for WB) (LI‐COR Biosciences, USA), HRP‐labeled Goat Anti‐Rabbit IgG (1:2000 for WB), HRP‐labeled Goat Anti‐Mouse IgG (Beyotime Biotechnology, China) (1:2000 for WB), ABflo 405‐conjugated Goat Anti‐Rabbit IgG (ABclonal, China) (1:5000 for IFA), Alexa Fluor 488‐conjugated goat anti‐mouse IgG (1:2000 for IFA), Alexa Fluor 594‐conjugated goat anti‐mouse IgG (1:2000 for IFA), Alexa Fluor 488‐conjugated goat anti‐rabbit IgG (1:2000 for IFA), Alexa Fluor 594‐conjugated goat anti‐rabbit IgG (1:2000 for IFA) (Fisher Scientific, USA). FITC‐conjugated Dolichos biflorus agglutinin (DBA) (Vector Laboratories, Burlingame, USA) (1:500 for IFA) was used for *Toxoplasma* cyst staining. IFA images were captured using an Olympus BX53 microscope (Olympus Life Science, Japan) equipped with an AxioCam 503 mono camera (Zeiss, Germany). Images were minimally adjusted for contrast and brightness using ZEN software (Carl Zeiss Inc., Germany). Western blots were scanned using an Amersham Typhoon 5 imager (GE Healthcare, UK) or a Tanon 5200 chemiluminescence imager (Tanon, China). Uncropped and unprocessed IFA and Western blot images are provided in the Supporting Information.

### Parasite Growth and Bradyzoite Differentiation Assays

Intracellular replication and plaque assays of tachyzoites were conducted to evaluate the growth and fitness of the transgenic strains, following previously described method.^[^ ^25^ ^]^ Each strain was analyzed in three independent experiments. To assess bradyzoite differentiation rates, purified parasites were allowed to invade HFF cells seeded on coverslips for 1 h under standard tachyzoite growth conditions. Non‐invaded parasites were washed away with PBS, and the cultures were incubated in alkaline medium (RPMI 1640 supplemented with 50 mM HEPES and 1% fetal bovine serum, pH 8.2) for 3 days at ambient CO₂, as described previously.^[^ ^26^ ^]^ The samples were then fixed with 4% paraformaldehyde and stained with FITC‐conjugated DBA and rabbit anti‐TgALD antibodies. Alexa Fluor 594‐conjugated goat anti‐rabbit IgG was used as the secondary antibody. The bradyzoite differentiation rate was determined by calculating the ratio of DBA⁺ vacuoles to ALD⁺ vacuoles. For the bradyzoite replication assay, parasites were cultured under alkaline conditions for 4 days and then allowed to invade HFF cells on coverslips for 1 h. After washing away non‐invaded parasites, the cultures were maintained in alkaline conditions for 36 h. Samples were fixed and stained with rabbit anti‐TgALD. Over 85 parasite vacuoles (PVs) were recorded for both the number of parasites within and the diameter. Each experiment was repeated three times independently.

### Virulence Tests in Mice

Virulence tests were conducted on 7‐8‐week‐old female Kunming mice, purchased from the Hubei Provincial Center for Disease Control. To assess the virulence and cyst formation, freshly harvested tachyzoites were intraperitoneally injected into the mice. Ten mice were used for each strain, and survival of infected mice was monitored daily for 30 days. After 30 days, the mice were euthanized, and brain tissues were collected. *Toxoplasma* cysts in the brains of surviving mice were quantified using DBA‐FITC staining, as described previously.^[^ ^27^ ^]^ All animal experiments and procedures were approved by the Ethics Committee of Huazhong Agricultural University (permit number: HZAUMO‐2019‐109).

### Delivery of HDAC3 to Target Gene Promoter by dCAS9

The pSRS34A‐dCAS9‐EGFP‐HDAC3‐U6‐sg*PAN* plasmid (15000 ng) was electroporated into freshly purified tachyzoites (10^7^ parasites) of the *ΔXII‐5*/pan‐HA strain, using previously described protocols.^[^ ^28^ ^]^ Transfection with the pSRS34A‐dCAS9‐EGFP‐U6‐sg*PAN* plasmid served as a negative control. Following transfection, parasites were allowed to invade HFF monolayers on coverslips for 4 h. After washing off non‐invaded parasites, the remaining parasites were cultured under standard tachyzoite conditions for 24 h. Subsequently, the samples were fixed with formaldehyde and stained with mouse anti‐HA and rabbit anti‐IMC1. Alexa‐594‐conjugated goat anti‐mouse IgG and ABflo 405‐conjugated Goat Anti‐Rabbit IgG were used as secondary antibodies. GFP positive parasites were subjected to fluorescent microscopy analyses using an Olympus BX53 microscope (Olympus Life Science, Japan) equipped with an AxioCam 503 mono camera (Zeiss, Germany). Images were acquired with the same exposure time across samples for the same channel. The red (HA signal) fluorescent intensity of imaged parasites was quantified using the ZEN software. Each experiment was performed in triplicate.

### RNA‐Seq and qRT‐PCR Analyses

Intracellular parasites were harvested, released by needle passage, and purified by filtration through membranes with 3 µm pore size to remove host cell debris. Total RNA was extracted from purified parasites using transZol Up reagent (TransGen Biotech, China) following the manufacturer's instructions. The RNA quality was assessed using an Agilent 2100 Bioanalyzer (Agilent, USA). Libraries for RNA sequencing were prepared using the TruSeq RNA Sample Prep Kit (Illumina, USA) and sequenced on a DNBSEQ‐T7 sequencer (MGI Tech Co., Ltd, China) with the PE150 model. Clean reads were mapped to the *T. gondii* ME49 genome (ToxoDB) using Hisat2 software.^[^ ^29^ ^]^ Transcript levels were calculated using the transcript per million (TPM) method, and differentially expressed genes were identified using DESeq2 v1.24.0 with a p‐value < 0.01 and a minimum fold change of 2. Each strain or condition was analyzed in triplicate, and raw data were deposited in the GEO database under accession number GSE277550. For quantitative RT‐PCR, RNA from the indicated strains was prepared as described above. Then, cDNA was synthesized from 800 ng of RNA using the HiScript III RT SuperMix for qPCR (+ gDNA wiper) kit (Vazyme Biotech, China). Quantitative PCR was performed using the QuantStudio 3 Real‐Time PCR System with ChamQ Universal SYBR qPCR Master Mix (Vazyme Biotech, China) and specific primers for selected genes (Tables S3, Supporting Information). Transcript levels of target genes were normalized to β‐tubulin mRNA levels.

### Co‐Immunoprecipitation (Co‐IP) and Mass Spectrometry

Freshly egressed tachyzoites of ME49, AP2XII‐5‐HA, ME49 Tir1, and AIP1‐mAID were purified by filtration through a 3 µm pore‐size membrane. The collected parasites (10⁸) were washed twice with chilled PBS and lysed in NP40 lysis buffer (150 mM NaCl, 1% NP‐40, 50 mM Tris pH 7.4) (Beyotime Biotechnology, China) for 30 min. After centrifugation at 5000 rpm for 10 min, the supernatants were used for Co‐IP. The supernatants were first incubated with protein G magnetic beads coupled with naive mouse or rabbit IgG at 4 °C for 1 h to remove parasite proteins bind to IgG or beads non‐specifically. The cleared supernatants were then incubated overnight at 4 °C with protein G beads coupled to either mouse anti‐HA or rabbit anti‐HDAC3 antibodies. Afterward, the beads were washed five times with chilled TBS to remove unbound proteins. Bound proteins were subjected to on‐beads trypsin digestion and mass spectrometry analyses for protein identification as described previously.^[^ ^30^ ^]^

### CUT&Tag and Data Analysis

CUT&Tag was used to identify HDAC3 occupancy in the genomes of ME49 and *ΔXII‐5* strains. Intracellular parasites were released by needle passage and purified through filtration with 3 µm pore‐size membranes. Approximately 5 × 10⁶ tachyzoites were collected and processed using the Hyperactive Universal CUT&Tag Assay Kit for Illumina (Vazyme Biotech, China), following the manufacturer's instructions. Rabbit anti‐TgHDAC3 was used as the primary antibody for immunoprecipitation. Side‐by‐side experiments using naïve rabbit IgG were included as controls. The resulting DNA was amplified for library construction using the TD202 TruePrep Index Kit V2 for Illumina (Vazyme Biotech, China). The libraries were enriched, quantified, and sequenced on a NovaSeq 6000 sequencer (Illumina) using the PE150 model. High‐throughput sequencing was conducted by Seqhealth Technology Co., LTD (Wuhan, China) and data analysis was conducted by Yingzi Gene Technology Co.,Ltd (Wuhan, China). The experiment was repeated twice independently. In brief, raw sequencing data were initially filtered using cutadapt (version 2.5) to remove low‐quality reads and trim adaptor‐contaminated sequences.^[^ ^31^ ^]^ Clean reads were mapped to the *T. gondii* reference genome (TGME49) using Bowtie2 (version 2.3.4.1) with default parameters.^[^ ^32^ ^]^ Library quality was assessed by calculating insert sizes using samtools (version 1.12) and analyzing read distribution around transcription start sites (TSS) with deepTools (version 3.5.1).^[^ ^33^ ^]^ Peak calling was performed using MACS2 (version 2.1.1).^[^ ^34^ ^]^ Bigwigs were generated using bigWigMerge and bedGraphToBigWig (version 4.11.1). Peaks were annotated using the ChIPseeker (version 1.32.1).^[^ ^35^ ^]^ Visualization of peaks in the genomic regions was performed using the IGV genome browser. The raw data have been deposited in the GEO database under the accession number GSE283441.

### Statistics and Reproducibility

All quantitative data were presented as mean ± standard deviation (SD), mean ± standard error of the mean (SEM), or median with interquartile range (IQR), as specified in the figure legends. Statistical analyses were conducted using Prism version 8.0 (GraphPad Software Inc., USA). Unpaired two‐tailed Student's t‐tests were applied for comparisons between two groups, and two‐way ANOVA with Tukey's multiple comparisons post‐tests was used for multiple group analyses, as detailed in the figure legends. Exact p‐values were displayed directly in the figures for clarity. Transcriptomic and CUT&Tag data were analyzed using bioinformatic tools described in the Methods section. Qualitative and quantitative experiments, such as IFA and Western blotting, were independently repeated at least twice with consistent results. Representative images were shown for these experiments.

## Conflict of Interest

The authors declare no conflict of interest.

## Author Contributions

L.X., F.F., and B.S. designed the study. L.X., J.Z., L.Z., F.F., and X.Y. performed the experiments. J.Z., X.Y., and H.T. contributed significantly to animal experiments. F.F. was involved in discussions and helped with the designs of the experiments. L.X. and B.S. performed the data analyses and wrote the manuscript. All authors read and approved of the final manuscript.

## Supporting information



Supporting Information

Supporting Information

Supporting Information

Supporting Information

## Data Availability

The data that support the findings of this study are openly available in RNA‐Seq data in the GEO database at https://www.ncbi.nlm.nih.gov/geo/query/acc.cgi?acc=GSE277550, reference number 277550 and in ChIP‐Seq database at https://www.ncbi.nlm.nih.gov/geo/query/acc.cgi?acc=GSE283441, reference number 283441.
