# Supplementary material for: A Novel Nuclear Protein Complex Controlling the Expression of Developmentally Regulated Genes in Toxoplasma Gondii
Source: Adv Sci (Weinh). 2024 Dec 24;12(7):2412000. doi: 10.1002/advs.202412000 (PMC11831446; doi:10.1002/advs.202412000)
Supplement: Supplementary file 1 — Supporting Information [file ADVS-12-2412000-s002.pdf]

ADVANCED SCIENCE
Open Access

Supporting Information

for *Adv. Sci.*, DOI 10.1002/advs.202412000

A Novel Nuclear Protein Complex Controlling the Expression of Developmentally Regulated Genes in *Toxoplasma Gondii*

*Lilan Xue, Jingwen Zhang, Lihong Zhang, Fuqiang Fan, Xiaoyan Yin, Hengrui Tian and Bang Shen**

Supporting Information

A Novel Nuclear Protein Complex Controlling the Expression of Developmentally Regulated Genes in *Toxoplasma gondii*

*Lilan Xue, Jingwen Zhang, Lihong Zhang, Fuqiang Fan, Xiaoyan Yin, Hengrui Tian, Bang Shen**

Lilan Xue, Jingwen Zhang, Lihong Zhang, Fuqiang Fan, Xiaoyan Yin, Hengrui Tian, Bang Shen
State Key Laboratory of Agricultural Microbiology, College of Veterinary Medicine, Huazhong Agricultural University. Wuhan 430070, P.R. China
E-mail: shenbang@mail.hzau.edu.cn

Bang Shen
Hubei Hongshan Laboratory, Wuhan 430070, P.R. China

Bang Shen
Shenzhen Institute of Nutrition and Health, Huazhong Agricultural University, Shenzhen 518000, P.R. China

Bang Shen
Shenzhen Branch, Guangdong Laboratory for Lingnan Modern Agriculture, Genome Analysis Laboratory of the Ministry of Agriculture, Agricultural Genomics Institute at Shenzhen, Chinese Academy of Agricultural Sciences, Shenzhen 518000, P.R. China

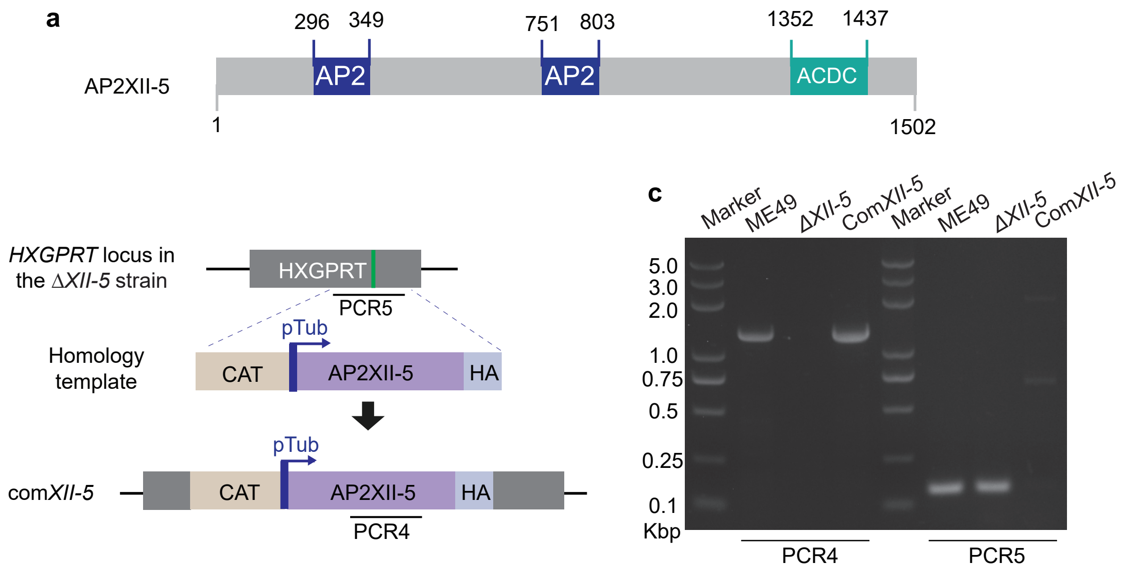

Figure S1. Construction of the comXII-5 strain. a) domain structures of TgAP2XII-5 predicted by SMART (http://smart.embl-heidelberg.de/). b) schematic representation of complementing TgAP2XII-5 at the *HXGPRT* locus of the *ΔXII-5* strain using CRISPR/Cas9 mediated homologous recombination. The green bar indicates the CRISPR targeting site. c) diagnostic PCRs for a representative clone of com*XII-5*.

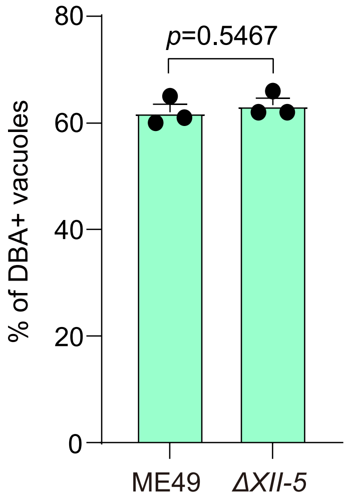

Figure S2. Deletion of AP2XII-5 does not affect the transition of tachyzoites to bradyzoites *in vitro*. The percentage of DBA positive PVs in indicated strains cultured in alkaline medium (pH = 8.2, ambient CO_2) for three days was graphed. Means ± SEM of n=3 independent experiments, each with three technical replicates, unpaired two-tailed Student’s t-test.

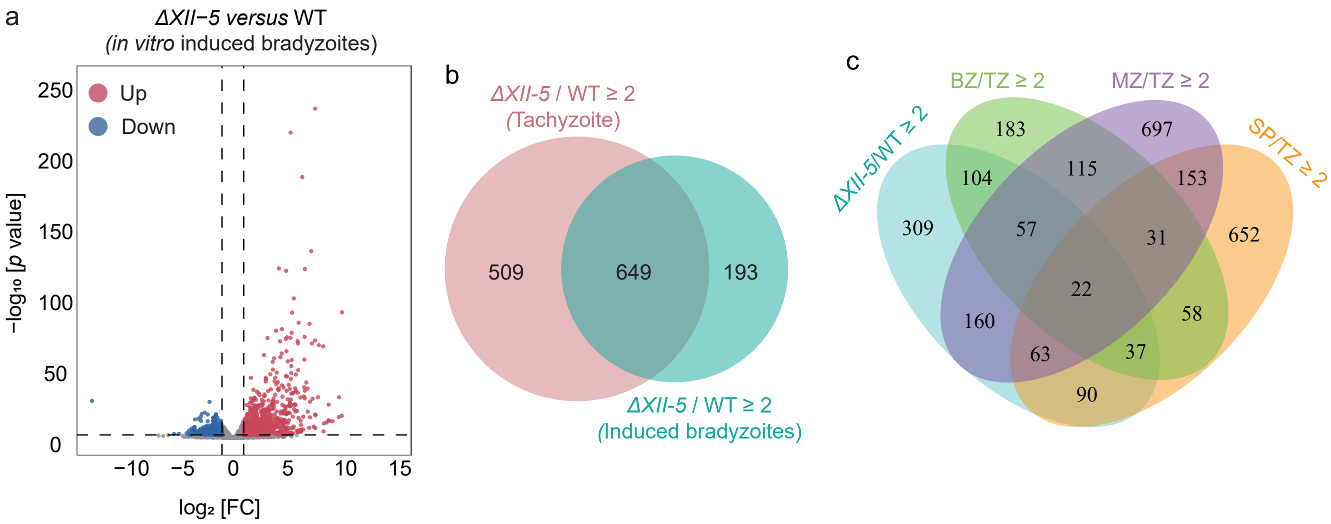

Figure S3. Gene expression changes caused by *TgAP2XII-5* deletion under bradyzoite-inducing conditions. a) Volcano plot showing differentially expressed genes between WT and *ΔXII-5* strains after alkaline induction, as determined by RNA-seq. Data from three biological replicates were plotted. Red and blue dots indicate the number of genes significantly up- and down-regulated after TgAP2XII-5 deletion (p-value < 0.01 and fold change (FC) ≥ 2 as cut-off thresholds), respectively. b) Venn diagram illustrating the overlap of genes that were significantly upregulated in the *ΔXII-5* mutant under tachyzoite and alkaline induced bradyzoite conditions. c) Venn diagram showing the overlap of genes significantly upregulated in *ΔXII-5* bradyzoites and those upregulated in wildtype bradyzoites (BZ), merozoites (MZ) or sporozoites (SP) compared to tachyzoites (TZ), as done in Figure 2b.

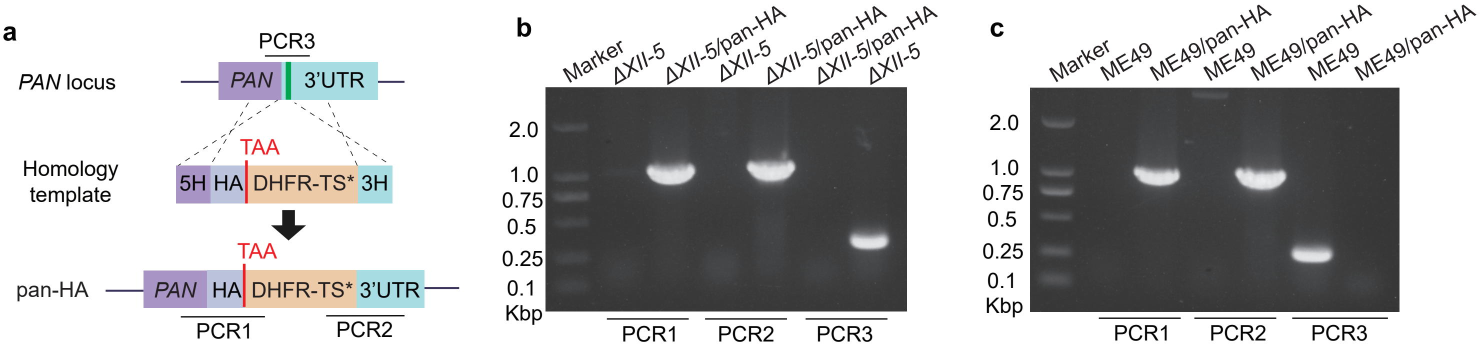

Figure S4. Construction of the pan-HA strains. a) Schematic representation of inserting an HA tag to the C-terminus of PAN at the endogenous *PAN* gene loci of the ME49 or ME49 *Δap2XII-5* strains, using CRISPR/Cas9 mediated homologous recombination. The green bar indicates the CRISPR targeting site. b, c) Diagnostic PCRs on representative clones of *ΔXII-5*/pan-HA and ME49/pan-HA.

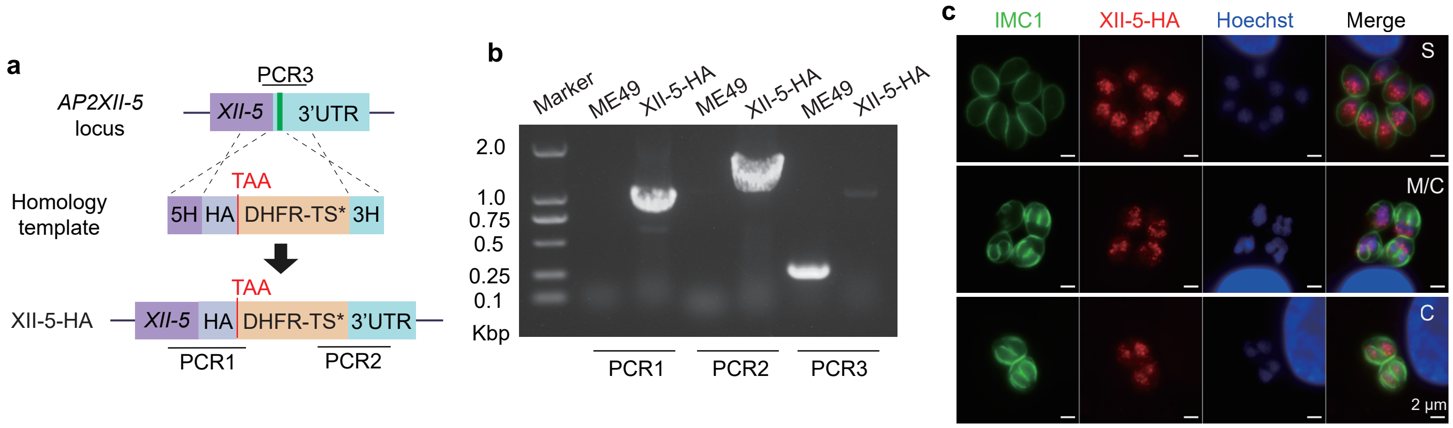

Figure S5. Construction of the AP2XII-5-HA strain. a) Schematic representation of inserting a spaghetti monster-HA tag (HA) to the C terminus of AP2XII-5 at the endogenous gene locus to construct the AP2XII-5-HA strain (XII-5-HA), using CRISPR/Cas9 assisted site specific insertion. The green bar indicates the CRISPR targeting site. b) Diagnostic PCRs on a representative clone of the AP2XII-5-HA strain. c) IFA checking the expression of *AP2XII-5* during the cell cycle of AP2XII-5-HA tachyzoites.

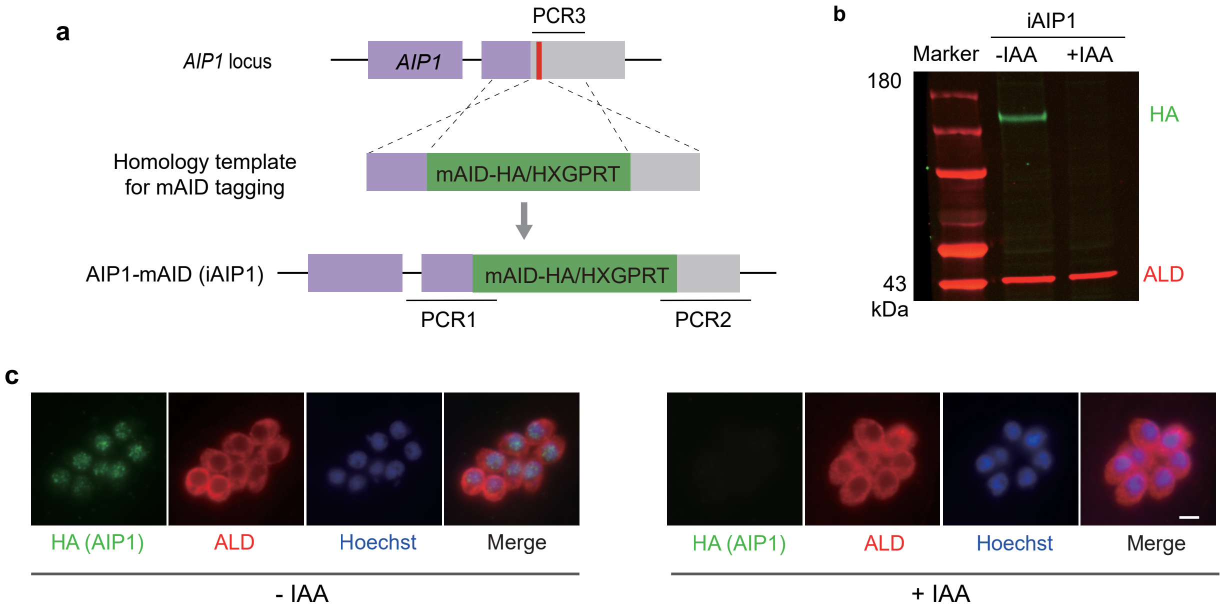

Figure S6. Construction of the AIP1-mAID strain. a) Schematic representation of inserting an mAID-HA tag to the C-terminus of endogenous AIP1 to construct the iAIP1 strain (AIP1-mAID). b, c) IFA and Western blotting on an iAIP1 clone to confirm the depletion of AIP1 (probed by anti-HA) after IAA treatment for 24 hours. Scale bar = 2 µm.

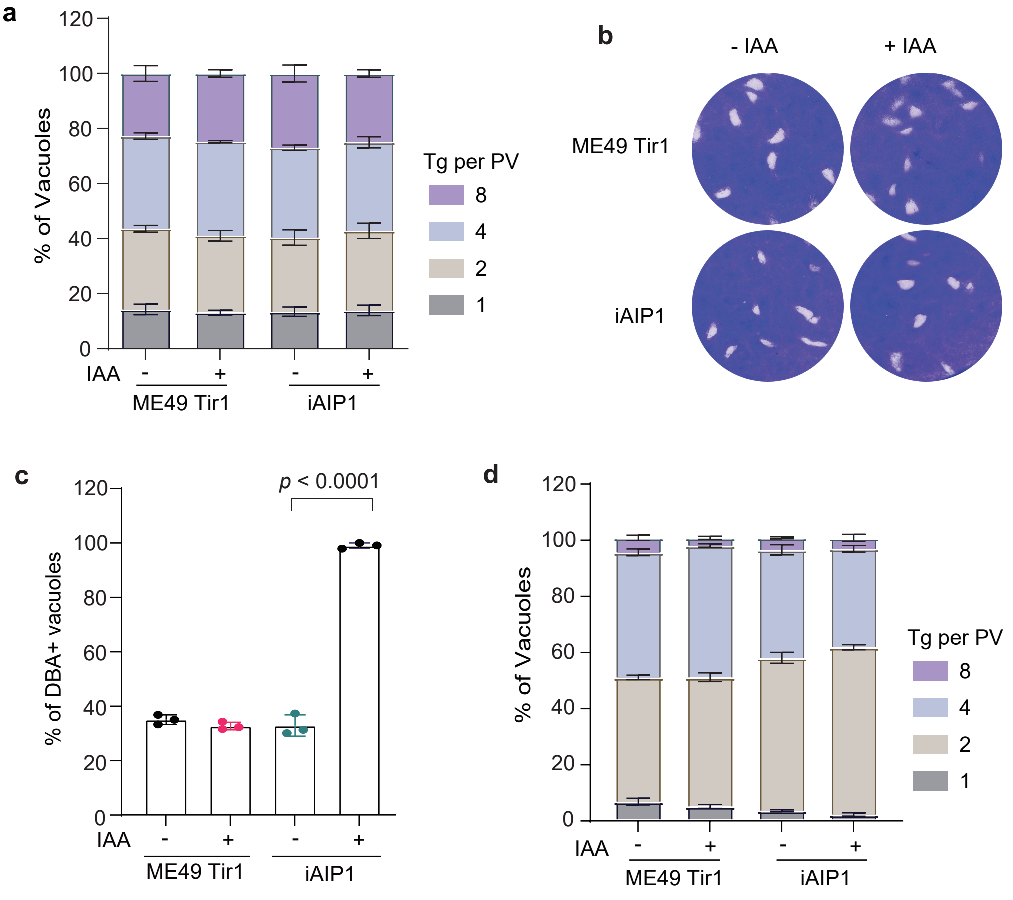

Figure S7. TgAIP1 is dispensable for tachyzoite growth i*n vitro*. a, b) The intracellular replication rates (a) and plaque formation efficiency (b) of indicated strains with or without IAA treatment under normal tachyzoite growth conditions. c) The percentage of DBA-positive PVs in indicated strains treated with or without IAA under alkaline growth conidtions (pH = 8.2, ambient CO_2). Means ± SEM of n=3 independent experiments, each with three technical replicates, unpaired two-tailed Student's t-test. d) The intracellular replication rates of indicated strains under alkaline growth conidtions. (a,d) Means ± SEM of n=3 independent experiments, each with three technical replicates, two-way ANOVA with Tukey's multiple comparisons post-tests.

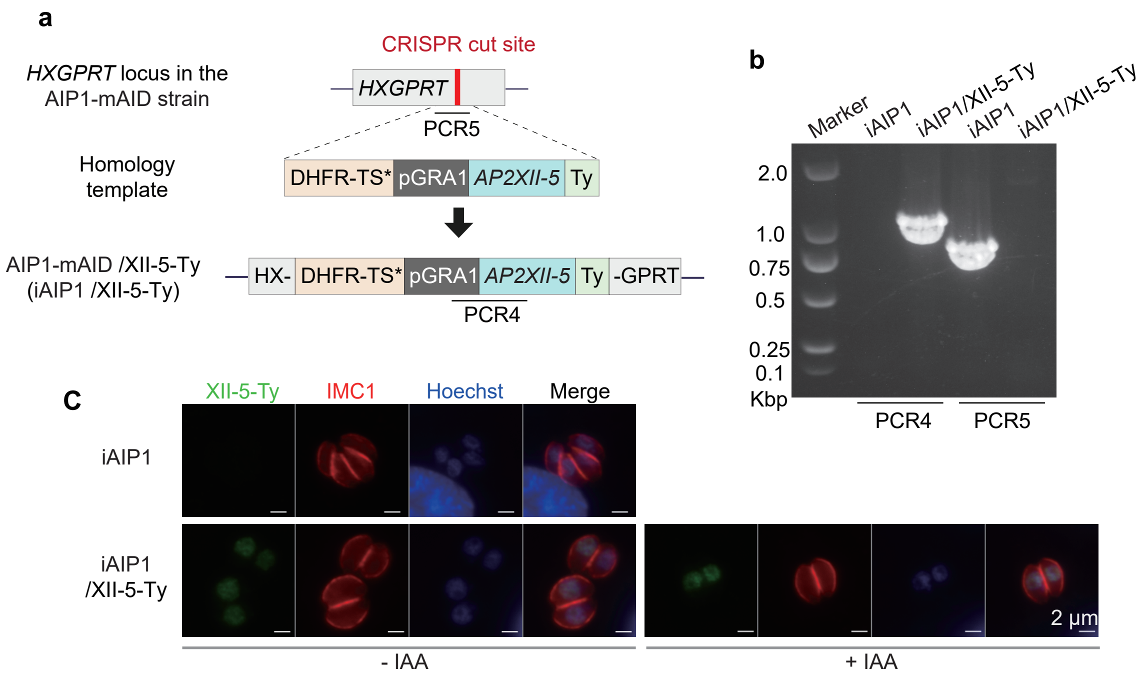

Figure S8. Construction of the AIP1-mAID/XII-5-Ty strain. a) Schematic representation of inserting an AP2*XII-5* expressing cassette driven by the GRA1 promoter into the *HXGPRT* locus of the AIP1-mAID strain to construct the AIP1-mAID/XII-5-Ty (iAIP1/XII-5-Ty) strain. b,c) Diagnostic PCRs and IFA on a representative clone of iAIP1/XII-5-Ty.

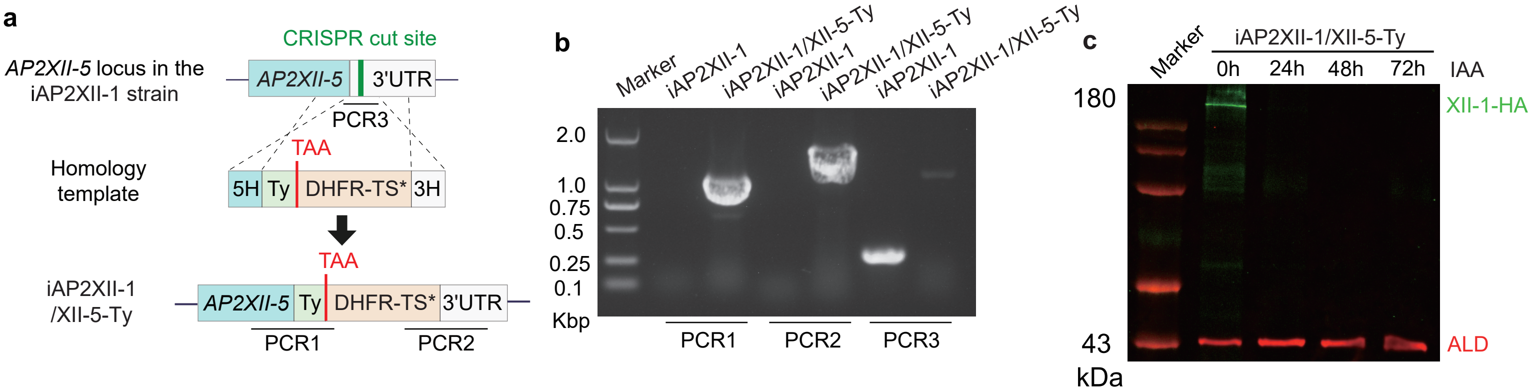

Figure S9. Construction of the iAP2XII-1/XII-5-Ty strain. a) Schematic representation of inserting a Ty tag to the C terminus of endegenous AP2XII-5 to generate the iAP2XII-1/XII-5-Ty strain. b) Diagnostic PCRs on a representative clone of *the* iAP2XII-1/XII-5-Ty strain. c) Western blotting checking the depletion of AP2XII-1 expression by IAA treatments (0/24/48/72 hours) in the iAP2XII-1/XII-5-Ty strain.

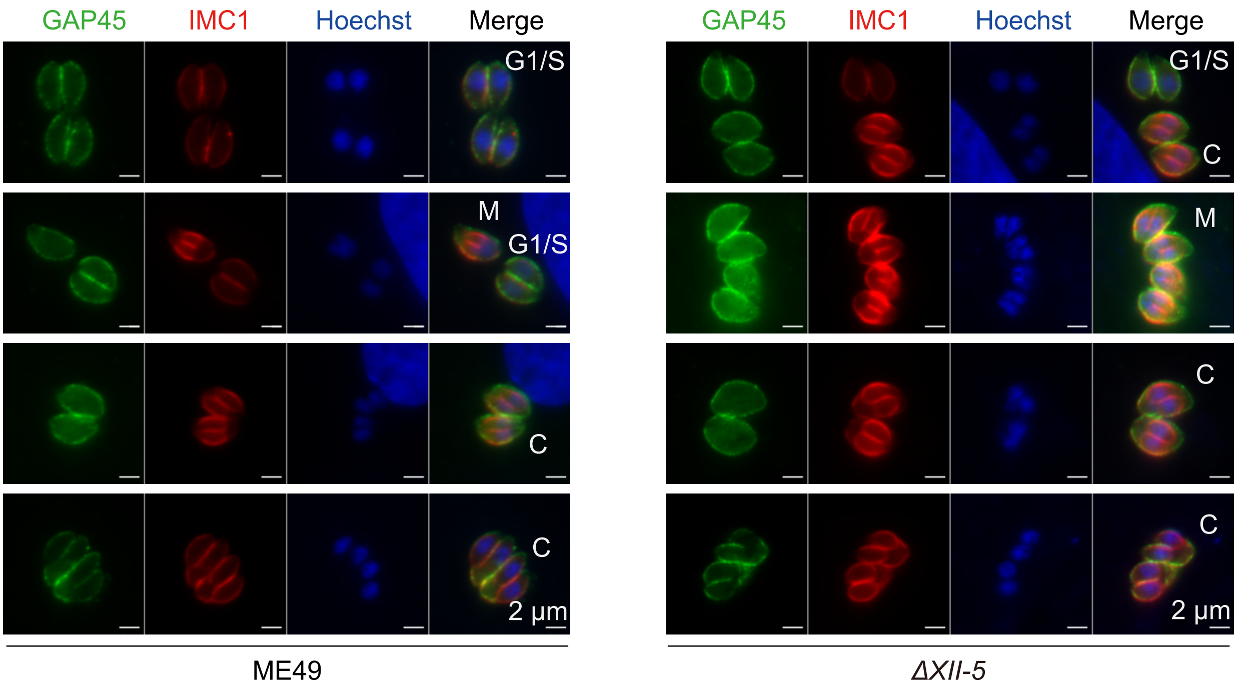

Figure S10. IFA checking the modes of parasite proliferation. The ME49 and *ΔXII-5* parasites were used to infect HFF monolayers and cultured for 24 hours under standard tachyzoite growth conditions. Subsequently the samples were fixed and stained with rabbit anti-IMC1 and mouse anti-GAP45. Scale bar represents 2 μm.
