# Supplementary material for: A Novel Nuclear Protein Complex Controlling the Expression of Developmentally Regulated Genes in Toxoplasma Gondii
Source: Adv Sci (Weinh). 2024 Dec 24;12(7):2412000. doi: 10.1002/advs.202412000 (PMC11831446; doi:10.1002/advs.202412000)
Supplement: Supplementary file 2 — Supporting Information [file ADVS-12-2412000-s003.docx]

**Supplementary** **Table S1-S3: Description of *T. gondii* Strains, Plasmids and Primers**

List of *T. gondii* parasite lines as well as plasmids used in this work. Primers and DNA synthesis construct used in this work are also charted in the table.

**Table S1. *T.gondii* strains used in this study**

| ***T. gondii* strain** | **Genotype** | **Use** | **Reference or source** |
| --- | --- | --- | --- |
| ME49 | ME49 | Parental strain | Dr. David Sibley |
| ME49 Tirl (ME49 *Δhxgprt*::Tir1) | ME49, *OsTir1-flag-CAT* | For the construction of AID tagged strains | Reference 1 |
| AP2XII-1-mAID (iAP2Ⅻ-1) | ME49, *OsTir1-flag-CAT*, ap*2XII-1-mAID-HA-hxgprt* | For the construction of iAP2XII-1/XII-5-Ty strain | Reference 2 |
| *ΔXII-5* (ME49 *Δap2XII-5*) | ME49, *ap2XII-5-dhfr* | Direct knockout of AP2XII-5 | This study |
| Com*XII-5* | ME49, *ap2XII-5-dhfr,* p*Tubulin-ap2XII-5-HA-CAT* | Complementation of the *ΔXII-5* | This study |
| AP2XII-5-HA | ME49, *ap2XII-5-10*HA-dhfr* | For Co-IP to identify proteins interacting with AP2XII-5 | This study |
| ME49/pan-HA | ME49, *TGME49_235390-10*HA HA-CAT* | To check the expression of PAN in ME49 strain | This study |
| *ΔXII-5/*pan-HA | ME49, *ap2XII-5-dhfr, TGME49_235390-10*HA HA-CAT* | To check the expression of PAN in *ΔXII-5* strain | This study |
| AIP1-mAID (iAIP1) | ME49, *OsTir1-flag-CAT, TGME49_314220-mAID-HA-hxgprt* | For IAA induced depletion of AIP1 in the ME49 Tir1 strain, as well as Co-IP to identify proteins interacting with AIP1 | This study |
| iAIP1/XII-5-Ty | ME49, *OsTir1-flag-CAT,* *TGME49_314220-mAID-HA-hxgprt*, p*GRA1-ap2XII-5-Ty-dhfr* | To check the overexpression of AP2XII-5 on the gene expression and protein interactions in iAIP1 strain | This study |
| iAP2XII-1/XII-5-Ty | ME49, *OsTir1-flag-CAT*, *ap2XII-1-mAID-HA-hxgprt*, *ap2XII-5-Ty-dhfr* | To check the expression of AP2XII-5 in iAP2XII-1 strain | This study |

**Table S2. Plasmids used in this study**

| **Plasmid name** | **Construction methods** | **Use** |
| --- | --- | --- |
| pDONR-DHFR | Reference 3 | Homology template for the DHFR* fragment amplification |
| pTUB1:YFP-mAID-3HA, DHFR-TS:HXGPRT | Reference 1 | Homology template for mAID-HXGPRT amplification |
| pUC19-smHA-3’UTR- DHFR | Reference 4 | Homology template for the construction of smHA tagged localization strains |
| pUC19-Ty-3’UTR-DHFR | Reference 4 | Homology template for the construction of Ty tagged localization strains |
| pUC19-smHA-3’UTR- CAT | Replace DHFR in pUC19-smHA-3’UTR- DHFR with CAT | Homology template for the construction of smHA tagged localization strains |
| pUC19-pTub:AP2X-4-HA~CAT | Reference 5 | Homology template for the construction of complemented strains |
| pSAG1::CAS9‐U6::sg*UPRT* | Reference 3 | Template for gene-specific CRISPR plasmid construction |
| pSAG1::Cas9‐U6: sg*HXGPRT*-intron | Reference 6 | For the construction of complemented strain and overexpression strain |
| pSAG1::Cas9‐U6::sg*AP2XII-5* | Replace sg*UPRT* in pSAG1::CAS9‐U6::sg*UPRT* with  sg*AP2XII-5* | CRISPR plasmid targeting AP2XII-5 CDS, for the construction of *ΔXII-5* strain. |
| pSAG1::Cas9‐U6::sg*AP2XII-5*-loc | Replace sg*UPRT* in pSAG1::CAS9‐U6::sg*UPRT* with  sg*AP2XII-5*-loc | CRISPR plasmid targeting AP2XII-5 3'UTR, for the construction of localization strain. |
| pSAG1::Cas9‐U6::sg*PAN*-loc | Replace sg*UPRT* in pSAG1::CAS9‐U6::sg*UPRT* with  sg*PAN*-loc | CRISPR plasmid targeting PAN-3'UTR, for the construction of localization strain. |
| pSAG1::Cas9‐U6::sg*AIP1* | Replace sg*UPRT* in pSAG1::CAS9‐U6::sg*UPRT* with  sg*AIP* | CRISPR plasmid targeting AIP1-3'UTR, for the construction of AIP1-mAID strain. |
| pUC19-AP2XII-5-DHFR | Insert the 5’ and 3’ homology arms of AP2XII-5 into pDONR-DHFR | Plasmids for the construction of *ΔXII-5* strain |
| pUC19-AP2XII-5-COMP | Replace AP2X-4 in pUC19-pTub:AP2X-4-HA~CAT with AP2XII-5 cds | Plasmids for the construction of ComXII-5 strain |
| pUC19-AP2XII-5-HA-DHFR | Insert the 5’ and 3’ homology arms of AP2XII-5 into pUC19-smHA-3’UTR- DHFR | Plasmids for the construction of AP2XII-5-HA strain |
| pUC19-AP2XII-5-Ty-DHFR | Insert the 5’ and 3’ homology arms of AP2XII-5 into pUC19-Ty-3’UTR- DHFR | Plasmids for the construction of iAP2XII-1/XII-5-Ty strain |
| pUC19-PAN-HA-CAT | Insert the 5’ and 3’ homology arms of PAN into pUC19-smHA-3’UTR- CAT | Plasmids for the construction of pan-HA strain |
| pUC19-AIP1-mAID-HXGPRT | Clone mAID-HXGPRT, 5’ and 3’ homology arms of AIP into pUC19 | Plasmids for the construction of AIP1-mAID strain |
| pUC19-pGRA1-ap2XII-5-Ty | Clone GRA1promoter, AP2XII-5 cds and Ty-DHFR fragment into pUC19 | Plasmids for the construction of iAIP1/XII-5-Ty strain |
| pSAG1::dCas9-U6::sg*UPRT* | Dr. David Sibley  dCas9(D10A and H840A) | Homology template for dCas9-U6::sg*UPRT* fragment amplification |
| pHR-SFFV-dCas9-BFP-KRAB | From addgene#46911 | Homology template for the KRAB fragment amplification |
| pSRS34A::dCAS9-EGFP-KRAB-U6::sg*UPRT* | dCAS9- EGFP-KRAB fusion driven by the SRS34A promoter, and sgRNA targeting *UPRT* | Homology template for dCAS9 plasmid construction |
| pSRS34A::dCAS9-EGFP-HDAC3-U6::sg*PAN* | Replace KRAB and sg*UPRT* in pSRS34A::dCAS9-EGFP-KRAB-U6::sg*UPRT* with the coding sequence of HDAC3 and sg*PAN* | Plasmids for dCAS9-HDAC3 assay |
| pSRS34A::dCAS9-EGFP-U6::sg*PAN* | Deleting HDAC3 in pSRS34A::dCAS9-EGFP-HDAC3-U6::sg*PAN* | Plasmids for dCAS9-HDAC3 assay |

**Table S3. Primers used in this study**

| **Primer name** | **Primer sequence 5' to 3'** | **Use** |
| --- | --- | --- |
| VETOR-F | GGCGTAATCATGGTCATAGC | Amplify the pUC19 backbone |
| VETOR-R | CTCGAATTCACTGGCCGTCG |  |
| AP2XII-5-gRNA-F | GCGAGTAGGCGGCAAACCACGTTTTAGAGCTAGAAATAGC | To construct the pSAG1::Cas9‐U6::sg*AP2XII-5* plasmid |
| AP2XII-5-gRNA-R | AACTTGACATCCCCATTTAC |  |
| AP2XII-5-5H-F | CGACGGCCAGTGAATTCGAGACAACGGGAGCTCTGAACAC | To construct the pUC19-AP2XII-5-DHFR plasmid |
| AP2XII-5-5H-R | GATGTCTTCTGCGCGGGTTGAGTGGCCGCTTCATCCGTC |  |
| AP2XII-5-3H-F | GCCACAAGTTCAGCGTGTCCACGCTGGGTTTCCGAAGTG |  |
| AP2XII-5-3H-R | GCTATGACCATGATTACGCCACTCCTCAGAAAGCGATGTG |  |
| Loxp-DHFR-loxp-F | CAACCCGCGCAGAAGACATC |  |
| Loxp-DHFR-loxp-F | GGACACGCTGAACTTGTGGC |  |
| iden-AP2XII-5-PCR1-F | ACAGTGACGAAAGGCTCGAC | Diagnostic PCRs for the identification of *ΔXII-5* strain construction |
| iden-AP2XII-5-PCR1-R | CGTGACCACGCCAAAGTAG |  |
| iden-AP2XII-5-PCR2-F | ATCACCAGCGCCATCGGTAG |  |
| iden-AP2XII-5-PCR2-R | ATGTCGCCAAAGTGGACGAAG |  |
| iden-AP2XII-5-PCR3-F | GAAGGAGCG*CAT*CAAAGAGG |  |
| iden-AP2XII-5-PCR3-R | GTCGATGTCTTCCTTCACCC |  |
| loc-AP2XII-5-gRNA-F | GTCAAATCGTGAGTAACGCC | To construct the pSAG1::Cas9‐U6::sg*AP2XII-5-*loc plasmid |
| loc-AP2XII-5-gRNA-R | CGACGGCCAGTGAATTCGAGGGGTCGAGCTACTATGGGAG |  |
| loc-AP2XII-5-5H-F | CGACGGCCAGTGAATTCGAGGGGTCGAGCTACTATGGGAG | To construct the pUC19-AP2XII-5-HA-DHFR and pUC19-AP2XII-5-Ty-DHFR plasmid |
| loc-AP2XII-5-Ty-5H-R | CTGGTTCGTGTGGACCTCCTTCGGAAACCCAGCGTC |  |
| loc-AP2XII-5-HA-5H-R | GGTACATCCTCGTCGAGCCCTTCGGAAACCCAGCGTC |  |
| loc-AP2XII-5-HA-3H-F | CCTGTTGAGAAAGCGGTGGCAAGAGGAATTTGACTGC |  |
| loc-AP2XII-5-Ty-3H-F | CACTTGCAGGATGAATTCCGCAAGAGGAATTTGACTGC |  |
| loc-AP2XII-5-3H-R | GCTATGACCATGATTACGCCCTGCACATCTGAGGTCTAC |  |
| iden-loc-AP2XII-5-PCR1-F | CAGGGCGTTCTACAGGGGC | Diagnostic PCRs for the identification of AP2XII-5-HA and iAP2XII-1/XII-5-Ty strain construction |
| iden-loc-AP2XII-5-Ty-PCR1-R | GCAGGTGAAGTAACATGGGG |  |
| iden-loc-AP2XII-5-HA-PCR1-R | GATATCCGGCGTAATCAGG |  |
| iden-loc-AP2XII-5-PCR2-F | TGACGCAGATGTGCGTGTAT |  |
| iden-loc-AP2XII-5-PCR2-R | ATGTCGCCAAAGTGGACGAAG |  |
| iden-loc-AP2XII-5-PCR3-F | GCCCAGCGCATTCTCTTCAG |  |
| iden-loc-AP2XII-5-PCR3-R | GATTCGCCTCAGCCAACTGG |  |
| comAP2XII-5-CDS-F | GATCTAAAATGGTGAGCAAGATGACGGATGAAGCGGCCACT | To construct the pUC19-AP2XII-5-COMP plasmid |
| comAP2XII-5-CDS-R | GGCATAATCTGGAACATCGTAAGGATACTTCGGAAACCCAGCGTCCTC |  |
| HA-TER-*CAT*-F | TATCCTTACGATGTTCCAGATTATGCCTAAATGCAG |  |
| HA-TER-*CAT*-R | CTTGCTCACCATTTTAGATCTA |  |
| iden-comAP2XII-5-PCR4-F | TGTTGTTCGTCTGTTCTCCG | Diagnostic PCRs for the identification of Com*XII-5* strain construction |
| iden-comAP2XII-5-PCR4-R | GTCCCACTTGCCTCGTCC |  |
| iden-comAP2XII-5-PCR5-F | CCCATGTATATCCCCGACAAC |  |
| iden-comAP2XII-5-PCR5-R | CCGTTCAAGTGCACTTACC |  |
| PAN-gRNA-F | GAAGTATCACTGGAATCTGCGTTTTAGAGCTAGAAATAGC | To construct the pSAG1::Cas9‐U6::sg*PAN*-loc plasmid |
| PAN-gRNA-R | GCAGATTCCAGTGATACTTCAACTTGACATCCCCATTTAC |  |
| PAN-5H-F | GGTATCGATAAGCTTCAGGAGGTCTTTTGTTGCTAC | To construct the pUC19-PAN-HA-CAT plasmid |
| PAN-5H-R | CATCCTCGTCGAGCCCGCAGCAGAGGCGCACGTCTTTG |  |
| PAN-3H-F | CACTAGTTCTAGAGCCCCGCAATGCGAAGAGCAAG |  |
| PAN-3H-R | CGCTCTAGAACTAGTTGGAATGGACACCGCCATCC |  |
| 10HA-*CAT*-F | GGCTCGACGAGGATGTACCC |  |
| 10HA-*CAT*-R | GCTCTAGAACTAGTGGATCC |  |
| iden-PAN-PCR1-F | AGAATGTCCCGCTACACAG | Diagnostic PCRs for the identification of PAN localization strain |
| iden-PAN-PCR1-R | GATATCCGGCGTAATCAGG |  |
| iden-PAN-PCR2-F | GGACCTGGCTGATGTTGATCG |  |
| iden-PAN-PCR2-R | GAGTGTTCGTGGACGGGAATG |  |
| iden-PAN-PCR3-F | ACTTACGTGGCTGCCACTTC |  |
| iden-PAN-PCR3-R | GGACTGACATGCCTCTCAGTTG |  |
| AIP1-gRNA-F | ttgcggtgagagactcaagaGTTTTAGAGCTAGAAATAGC | To construct the pSAG1::Cas9‐U6::sg*AIP* plasmid |
| AIP1-gRNA-R | tcttgagtctctcaccgcaaAACTTGACATCCCCATTTAC |  |
| AIP1-5H-F1 | GTGCACCATATGAAGTGTGTTGAAATCTGATTTTGGC | To construct the pUC19-AIP1-mAID-HXGPRT plasmid |
| AIP1-5H-R1 | TCACCATCCTAGGTGTCTGTCGAGAGAACGCCC |  |
| AIP1-mini-F2 | ACAGACACCTAGGATGGTGAGCGCTAGC |  |
| AIP1-mini-R2 | ACTTCTCAGGCATGCCCATTCGCCATTCAGGC |  |
| AIP1-3H-F3 | AATGGGCATGCCTGAGAAGTTCACTAGGAA |  |
| AIP1-3H-R3 | GGATCCCCGGGTACCTTGCTTATCGAAGAAACAACGCA |  |
| iden-AIP1-PCR1-F | aggtcaagagccgttcag | Diagnostic PCRs for the identification of AIP1-mAID clones |
| iden-AIP1-PCR1-R | GGCTTAGCGGGATCTTTAGG |  |
| iden-AIP1-PCR2-F | CCTGGCGTTACCCAACTTAATC |  |
| iden-AIP1-PCR2-R | acgttggcatccagaaagactc |  |
| iden-AIP1-PCR3-F | GGCAGTTCGTGTTGCCAGTC |  |
| iden-AIP1-PCR3-R | tctcaccgcaagccgatgtc |  |
| OE-pGRA1-F | ATCGATAAGCTAGAGCTTAGTAGTTCAGCGCTGTGACGC | To construct the pUC19-pGRA1-ap2XII-5-Ty plasmid |
| OE-pGRA1-R | CGCTTCATCCGTCATCAGCCACCAAACAACACAAGC |  |
| OE-XII-5-F | GTGTTGTTTGGTGGCTGATGACGGATGAAGCGGCC |  |
| OE-XII-5-R | TCCTGGTTCGTGTGGACCTCCTTCGGAAACCCAGCGTCC |  |
| OE-Ty-DHFR-F | GAGGTCCACACGAACCAGGA |  |
| OE-Ty-DHFR-R | AAGCTCTAGCTTATCGATAC |  |
| iden-OEXII-5-PCR4-F | TTGGGCAGTCGGTAAAGC | Diagnostic PCRs for the identification of iAIP1/XII-5-Ty clones |
| iden-OEXII-5-PCR4-R | GCTTCGCGTTGTACGATG |  |
| dCAS9-HDAC3-F | agtcactaactgcctggtcCATGGCGCTCAGTGCGCTG | To construct the pSRS34A-dCAS9-EGFP-HDAC3-U6-sg*PAN* plasmid |
| dCAS9-HDAC3-R | caacggtgattaattaactaGATCGGAACCTTTTGGTCTCTG |  |
| dCAS9-vector-F | TAGTTAATTAATCACCGTTGTGCTCA |  |
| dCAS0-vector-R | GGACCAGGCAGTTAGTGACTTAGC |  |
| sgPAN661-F | GAATCGGCGTCTTTCATGGAGTTTTAGAGCTAGAAATAGC |  |
| sgPAN661-R | TCCATGAAAGACGCCGATTCAACTTGACATCCCCATTTAC |  |
| dCAS9-GFP-F | CACTAACTGCCTGGTCCTAGTTAATTAATCACCGTTGTGC | To construct the pSRS34A::dCAS9-EGFP-U6::sg*PAN* plasmid |
| dCAS9-GFP-R | CGGTGATTAATTAACTAGGACCAGGCAGTTAGTGACTTAG |  |
| 235390-RT-F | TCCTCTGGCTCAACACAAA | qRT-PCR for mRNA abundance detect |
| 235390-RT-R | GCACCATTTCTGCTGTCTAC |  |
| 219828-RT-F | ACCCAAGAAGCCTCTTTCG |  |
| 219828-RT-R | GCTTAGCTTTGCCCATTCC |  |
| 294600-RT-F | TGTTGCAGAGGAAGCTTACG |  |
| 294600-RT-R | CGTCTCAGCTTCTTCGAATG |  |
| 311710-RT-F | TCGGTCTTACTTCTCCCTAC |  |
| 311710-RT-R | ATGGCGTCTTTAGGTGACTC |  |

Reference*

[1] K. M. Brown, S. Long, L. D. Sibley, *mBio* **2017**, *8* (3), <https://doi.org/10.1128/mBio.00375-17>.

[2] F. Fan, L. Xue, X. Yin, N. Gupta, B. Shen, *mBio* **2023**, *14* (5), e0178523, <https://doi.org/10.1128/mbio.01785-23>.

[3] B. Shen, K. M. Brown, T. D. Lee, L. D. Sibley, *mBio* **2014**, *5* (3), e01114, <https://doi.org/10.1128/mBio.01114-14>.

[4] Z. Niu, S. Ye, J. Liu, M. Lyu, L. Xue, M. Li, C. Lyu, J. Zhao, B. Shen, *PLoS pathogens* **2022**, *18* (11), e1011009, <https://doi.org/10.1371/journal.ppat.1011009>.

[5] J. Zhang, F. Fan, L. Zhang, B. Shen, *Microbiology spectrum* **2022**, *10* (4), e0012022, <https://doi.org/10.1128/spectrum.00120-22>.

[6] J. Yang, X. Yang, A. Liu, Y. Li, Z. Niu, C. Lyu, X. Liang, N. Xia, J. Cui, M. Li, P. Wu, C. Peng, B. Shen, *Cellular and molecular life sciences : CMLS* **2022**, *79* (10), 532, <https://doi.org/10.1007/s00018-022-04556-z>.
